# Effect on Nutritional and Functional Characteristics by Encapsulating *Rose canina* Powder in Enriched Corn Extrudates

**DOI:** 10.3390/foods10102401

**Published:** 2021-10-11

**Authors:** Marta Igual, Maria Simona Chiş, Adriana Păucean, Dan Cristian Vodnar, Floricuța Ranga, Tania Mihăiescu, Anamaria Iulia Török, Anca Fărcaș, Javier Martínez-Monzó, Purificación García-Segovia

**Affiliations:** 1 Food Investigation and Innovation Group, Food Technology Department, Universitat Politècnica de València, Camino de Vera s/n, 46022 Valencia, Spain; marigra@upvnet.upv.es (M.I.); xmartine@tal.upv.es (J.M.-M.); pugarse@tal.upv.es (P.G.-S.); 2 Department of Food Engineering, Faculty of Food Science and Technology, University of Agricultural Sciences and Veterinary Medicine of Cluj-Napoca, 400372 Cluj-Napoca, Romania; adriana.paucean@usamvcluj.ro (A.P.); anca.farcas@usamvcluj.ro (A.F.); 3 Faculty of Food Science and Technology, Institute of Life Sciences, University of Agricultural Sciences and Veterinary Medicine of Cluj-Napoca, 400372 Cluj-Napoca, Romania; dan.vodnar@usamvcluj.ro (D.C.V.); floricuta.ranga@usamvcluj.ro (F.R.); 4 Department of Environment and Plant Protection, Faculty of Agriculture, University of Agricultural Sciences and Veterinary Medicine of Cluj-Napoca, 400372 Cluj-Napoca, Romania; tania.mihaiescu@usamvcluj.ro; 5 NCDO-INOE 2000 Research Institute for Analytical Instrumentation, 67 Donath Street, 400293 Cluj-Napoca, Romania; iulia.torok@icia.ro

**Keywords:** biopolymers, *Rosa canina*, corn extrudates

## Abstract

Wild *Rose canina* fruit represents a rich source of bioactive compounds such as minerals, phenolic compounds, vitamins, carotenoids, folate, and antioxidant activity that still needs to be further exploited. Thus, this study aimed to use wild *Rosa canina* fruit encapsulated powder with different biopolymers aiming to manufacture ready-to-eat products, such as corn extrudates. To achieve this goal, extrudate physicochemical characteristics, such as water content (x_w_), water activity (a_w_), water absorption index (WAI), water solubility index (WSI), swelling index (SWE), hygroscopicity (Hy), expansion index (SEI), bulk density (ρ_b_), porosity (ε), textural, optical; nutritional; and functional analysis (phenolic acids, flavonoids, ascorbic and dehydroascorbic acids, vitamin C, carotenoids, folates, antioxidant activity, and minerals) were determined. Results highlighted that 4 and 8% addition of wild *Rose canina* fruit encapsulated powder could be successfully used in the corn extrudates, showing the positive influence on its nutritional and functional value. Strong positive Pearson correlations were identified between antioxidant capacity and total flavonoids, carotenoids, folates, and vitamin C of mixtures and extrudates Minerals increased their amount during the extrusion process, reaching the highest values at an addition of 8% rosehip encapsulated with pea protein biopolymer. Furthermore, from the biopolymers used in the present study, pea protein powder exhibited the highest protection on the analyzed bioactive compounds against the extrusion process.

## 1. Introduction

The market for ready-to-eat products is constantly developing in the context of the social habits of the modern consumer. Furthermore, because of their sensorial features such as taste, texture, palatability, and appearance, ready-to-eat products are attractive to consumers [1,2].

The most common technique of producing ready-to-eat foods is extrusion cooking, the process of pressing the powder mixture through a die with a specific shape, which causes the material [3]. A wide range of food products could be produced with this technique, such as crisp expanded snacks, breakfast cereals, instant soups, meat analogs, and sports foods [4]. The main raw materials used for snack production by extrusion are cereals (e.g., corn and rice) due to their good expansion properties and enhancement of starch digestibility caused by gelatinization [2,4]. A recent review highlighted the possibility of incorporating pseudo-cereals, fruits, vegetables, legumes, pulses, oilseeds, roots, and tubers, nuts, and seeds to obtain value-added products [3].

Wild rose (*Rosa canina* L.) is a native shrub that belongs to the *Rosaceae* family and is widespread in northern Europe, Asia, Middle East, and North America. For centuries, the pseudo-fruits of *Rosa canina* (rose hips) are recognized as valuable food and medicine constituents due to their notable content of pro-health compounds [5]. The beneficial health effects are related to their rich content in flavonoids, carotenoids, fatty acids, vitamins, especially vitamin C, and folate [6].

A large body of literature has reported on the antioxidant, anti-inflammatory activities, antibacterial, antimutagenic, anti-diabetic, and anti-cancerogenic effects, as well as the capacity to balance the lipids level as well as the glucose content in blood [5,7,8,9,10,11]. The studies demonstrated that the compounds with antioxidant activity are polyphenols, vitamins C, E, B, and carotenoids known with synergistic effects [6,12].

The polyphenolic compounds include flavonoids: anthocyanins, procyanidins, catechin, quercetin, phenolic acids—gallic and ellagic acids—kaempferol, apigenin, and resveratrol, whereas the carotenoids found in rose hips are lycopene, β-carotene, and zeaxanthin [7]. Studies also reported high content of vitamin C and polyphenolics, especially quercetin, ellagic acid, gallic acid, and catechin in rose hips.

The addition of fruits into extruded products is considered a valuable way for the enhancement of the nutritional characteristics of extruded foods. However, the action of high-pressure temperature, combined with shear during extrusion leads to loss of sensitive nutritional compounds. In addition, several physicochemical factors could inactivate the bioactive compounds during food processing, storage, and digestion.

Thus, recent findings have investigated the ability of some polymers to allow the formulation of extruded foods with ingredients that require protection or stability; this approach extrusion could become a promising practice to deliver bioactive nutrients [3]. Moreover, recent findings report the use of biopolymers—comprising polysaccharides and proteins—to protect and deliver bioactive compounds [13].

Biopolymers act by forming matrices with bioactive compounds through molecular entanglement. Generally, it is considered that polysaccharides entrap the bioactives by surrounding the core, while proteins act like emulsifiers, swelling, and solubilization agents [14]. The most used polysaccharides are starch, dextrin, maltodextrin, cyclodextrin, alginate, pectin, cellulose, and gum. For proteins, non-allergic plant proteins extracted from pulses, cereals, or oilseeds using eco-friendly techniques have gained attention recently [15]. Several studies have reported the use of maltodextrin to encapsulate orange turpentine, tocopherol, and ascorbic acid using extrusion [16,17]. It was also reported that starch, mannose, and cyclodextrins could be used for the same purpose. However, maltodextrin is usually preferred because it is cheaper and ready to use [13]. Further, soy, pea, and chickpea protein-based matrices successfully encapsulated lycopene and folate [18,19].

In the present study, biopolymers such as maltodextrin, pea protein powder, beta-cyclodextrin and resistant maltodextrin were used in rosehip manufacturing before the extrusion process. Rosehip addition in the final extrudates was 4% and 8% respectively. Thus, this study aims to evaluate the impact of enrichment with two quantities of *Rosa canina* rosehip powder encapsulated with different biopolymers, on nutritive and functional value, physicochemical properties, and extrusion parameters of extruded corn snacks.

## 2. Materials and Methods

### 2.1. Standards and Reagents

Acid ascorbic standard, carotene, and folic acid standards were purchased from Sigma-Aldrich (Steinheim, Germany) with a purity level ≥99.9%. H_2_O_2_, HNO_3_, chlorogenic acid (>98% HPLC), rutin (>99% HPLC), gallic acid (>99% HPLC), and Multi-elemental solutions of 1000 mg L^−1^ ICP Standard Certipur^®^ were achieved from Merk, (Darmstadt, Germany). All other chemicals were purchased from the same supplier. A Millipore Direct-Q UV system from Merck (Darmstadt, Germany) was used to purified water.

### 2.2. Raw Materials

Corn grits (CM) were supplied by Maicerías Españolas S.L. (Valencia, Spain). Rosehip (*R. canina*) fruits were manually harvested in Aldehuela (Teruel, Spain) in September 2020. Maltodextrin (GLUCIDEX^®^ 12) (MD), pea protein powder (Nutralys^®^ S85F) (PP), and beta-cyclodextrin (KLEPTOSE^®^) (CD) were supplied by Roquette S.L. (Valencia, Spain). Resistant maltodextrin (Fibersol-2^®^) (RMD) was purchased from ADM/Matsutani, LLC (Decatur, IL, USA).

### 2.3. Rosehip Powder Manufacturing

Rose hips (1000 g) were washed with distilled water and homogenized in a Thermomix (TM 21, Vorwerk, Valencia, Spain) for 1 min at 5200 rpm. Then, distilled water (1000 g) was added and newly re-homogenized for 5 min at 5200 rpm. The mixture was filtered using a sieve (light of mesh diameter 1 mm, Cisa 029077). Four different formulations were prepared by adding 10 g of MD, RMD, PP, or CD to 90 g of the filtered mixture. Moreover, a control sample (R) without biopolymers was prepared. The formulated rosehip and control purees were then freeze-dried. A puree layer (0.5 cm thickness) was placed in a standardized aluminum plate with the following dimensions: 15 cm diameter and 5 cm height. Consecutively, samples were stored at −45 °C (Vertical Freezer, CVF450/45, Ing. Climas, Barcelona, Spain) for 24 h before being dried in a Lioalfa-6 Lyophyliser (Telstar, Spain) at 2600 Pa and −56.6 °C for 48 h. The freeze-dried samples were ground in a grinder (Minimoka, Taurus, Lleida, Spain) to obtain a free-flowing powder. Therefore, the powdered products obtained from rosehip were R (rosehip), MDR (maltodextrin rosehip), RMDR (resistant maltodextrin rosehip), PPR (pea protein rosehip), and CDR (cyclodextrin rosehip).

### 2.4. Formulations and Extrusion Processing

CM was mixed manually using a whisk, with two quantities (4 or 8%, 4R, and 8R) of obtained rosehip powders (R, MDR, RMDR, PPR, and CDR) to produce the extrusion mixtures (R4M, MDR4M, RMDR4M, PPR4M, CDR4M, R8M, MDR8M, RMDR8M, PPR8M, and CDR8M). The extrudates were coded as follows: R4M—extrudate with 4% rosehip addition, MDR4M—extrudate with maltodextrin rosehip 4% addition, RMDR4M—extrudate with resistant maltodextrin 4% rosehip addition, PPR4M—extrudate with pea protein 4% rosehip addition, CDR4M—extrudate with cyclodextrin 4% rosehip addition, R8M—extrudate with 8% rosehip addition, MDR8M—extrudate with maltodextrin rosehip 8% addition, RMDR8M—extrudate with resistant maltodextrin 8% rosehip addition, PPR8M—extrudate with pea protein 4% rosehip addition and CDR8M—extrudate with cyclodextrin 8% rosehip addition).

A single-screw laboratory extruder (Kompaktextruder KE 19/25; Brabender, Duisburg, Germany) was used for extrusion process. The total material extrusion amount was 200 g. The operating conditions were: 3:1 compression ratio; 18 rpm of dosing speed (3.4 kg/h); 150 rpm screw rotation; 25, 70, 170, and 175 °C of temperature in barrel sections and 3 mm of the nozzle. The calculated specific mechanical energy of the corn extrusion ranged from 950 to 1100 kJ/kg. The pressure measured on the extruder head ranged between 92 and 127 bar. Extrudates were cooled at ambient temperature and sealed in plastic bags for further analysis. The extrudates obtained were: R4E, MDR4E, RMDR4E, PPR4E, CDR4E, R8E, MDR8E, RMDR8E, PPR8E, and CDR8E.

### 2.5. Analysis

#### 2.5.1. Water Content and Water Activity

Water content (x_w_) (g water/100 g sample) of mixtures and extruded was determined according to AOAC [20] in triplicate. Water activity (a_w_) of the extruded samples was measured by the AquaLab PRE LabFerrer equipment (Pullman, Washington, DC, USA).

#### 2.5.2. Expansion Index (SEI), Bulk Density (ρ_b_) and Porosity (ε)

The diameter of samples was measured for each sample with an electronic Vernier caliper (Comecta S.A., Barcelona, Spain). The surface expansion index of the die (SEI) was calculated as the quotient between the square of the measured diameters and the square of the die diameter [21]. ρ_b_ was calculated from the height and diameter of cylinders (extrudates samples) and then their weight was measured [21]. The porosity (ε) was calculated according to García-Segovia et al. [21]. 

#### 2.5.3. Water Absorption Index (WAI), Water Solubility Index (WSI), and Swelling Index (SWE)

WAI and WSI were determined by the method of Singh and Smith [22] and calculated according to Uribe-Wandurraga et al. [23]. SWE was measured using the bed volume technique expressed as mm of swollen sample per g of the dry initial sample [24].

#### 2.5.4. Hygroscopicity (Hy)

Samples were placed in a petri dish at 25 °C, in an airtight plastic container containing Na_2_SO_4_ saturated solution (81% relative humidity). Initially and after 7 days each sample was weighed and the Hy was expressed as g of water gained per 100 g dry solids [25]. 

#### 2.5.5. Texture

The average puncturing force (F_p_), the average specific force of structural ruptures (F_s_), the spatial frequency of structural ruptures (N_sr_), and crispness work (W_c_), and the number of peaks (N_o_) were obtained from the force–time curve of puncture test [26,27,28]. It was measured using a TA-XT2 Texture Analyzer (Stable Micro Systems Ltd., Godalming, UK) and software, Texture Exponent (version 6.1.12.0, Stable Micro Systems Ltd., Godalming, UK).

#### 2.5.6. Optical Properties

Both translucency [29] and CIE*L*a*b* color coordinates were determined following the methodology described by García-Segovia et al. [21], considering standard light source D65 and a standard observer 10° (Minolta spectrophotometer CM-3600d, Tokyo, Japan). Measurements of the extruded samples were taken 10 times. The total color differences of mixtures or extrudates with encapsulated rosehip powder (ΔE_1_) were calculated for the control sample. To evaluate the color changes of the mixtures because of extrusion, the total color difference (ΔE_2_) was calculated between each mixture and extruded at the same encapsulated rosehip powder addition. 

#### 2.5.7. Samples Extraction Assisted by Ultrasounds

For the phenolic compounds, a total amount of 0.5 g of each sample was homogenized with 2 mL of methanol and 1% HCl. The samples were mixed for 1 min with a vortex (Heidolph, Schwabach, Germany). Furthermore, the samples were sonicated for 30 min in an ultrasonic bath (Elmasonic E15H, Elma, Singen, Germany) and centrifugated (4000× *g* for 10 min) with a centrifuge Eppendorf 5804 (Eppendorf, Hamburg, Germany). Afterward, samples were filtered through a 0.45 µm nylon filter (Millipore, Merck KGaA, Darmstadt, Germany) and injected into the HPLC system, according to our previous work, Igual et al. [30].

Ultrasound-assisted extraction for carotenoids was made according to Szabo et al. [31]. Shortly, 0.5 g of each sample was mixed with 5 mL mixture of methanol/ethyl:acetate/petroleum:ether (1:1:1, *v*/*v*/*v*), sonicated, and centrifuged (5 min, 8000× *g*) using Eppendorf 5804 centrifuge (Eppendorf, Hamburg, Germany). After, a separation funnel was used for the supernatant collection. To obtain the sample’s complete discoloration, the pellet was re-extracted three more times, following the same procedure. Sodium chloride solution (15%) was used to wash the collected extracts; then the organic phase was dried, and the solvent was eliminated by using an evaporator (Rotavapor R-124, Buchi, Flawil, Switzerland).

Ascorbic (AA) and dehydroascorbic (DHA) acids were analyzed by using ultrasound-assisted extraction. Shortly, 0.5 g of sample was mixed with 3 mL of 3% H_3_PO_4_ and 8% acetic acid in an aqueous solution, sonicated for 30 min at 20 °C (Elmasonic E15H) and centrifugated at 4000× *g*, 10 min, 4 °C (Eppendorf). Afterward, after filtration, 20 μL of each sample was injected into an HPLC system, according to our previous work [30].

For the folate extraction, 1 g of sample was mixed with 5 mL phosphate buffer (pH = 7), sonicated in the ultrasonic bath for 30 min, centrifuged at 4000× *g*, for 10 min and 24 °C, filtered, and injected in the HPLC system, according to Igual et al. [30].

#### 2.5.8. Phenolic Compounds Analysis through HPLC-DAD-ESI-MS (High-Performance Liquid Chromatography–Diode Array Detection–Electro-Spray Ionization Mass Spectrometry)

The method described in our previous work Igual et al. [30] was used for the identification and quantification of phenolic compounds. Analysis was determined by using an HP-1200 liquid chromatograph equipped with a quaternary pump, autosampler, DAD detector, and MS-6110 single-quadrupole API-electrospray detector (Agilent-Technologies, Santa Clara, CA, USA). The positive ionization mode was applied to detect the phenolic compounds; different fragmentor in the range 50–100 V, was applied. Eclipse XDB-C18 columns were used (5 μm; 4.6 × 150 mm) from Agilent and the mobile phase was (A) water acidified by acetic acid 0.1% (*v*/*v*) and (B) acetonitrile acidified by acetic acid 0.1% (99:1, *v*/*v*) with the flow rate was 0.5 mL/min, following the elution program described by Dulf et al. [32]. The ESI (+) module was applied for MS fragmentation in the same parameters described above with a scan range of 100–1200 *m*/*z*. 

Wavelengths of λ = 254, 280, and 340 nm were used for recording the chromatograms, and data acquisition was registered with the Agilent ChemStation software (Rev B.04.02 SP1, Palo Alto, CA, USA). The phenolics identification was based on UV-visible spectra, time retention, mass spectra, and chromatography with authentic standards (when available). 

The quantification of flavonoids (flavones and isoflavones) was realized based on the rutin standard calibration curve (y = 26.935x − 33.784, r^2^ = 0.9981) with a concentration ranged between 10–100 μg/mL and expressed as equivalents of rutin (µg rutin/g_dry weight_), hydroxybenzoic acid was quantified using the calibration curve performed with gallic acid (y = 33.624x + 30.68, r^2^ = 0.9978) on the concentration range of 1–100 μg/mL and expressed as gallic acid equivalents (µg gallic acid/g_dry weight_), and hydroxycinnamic acids (caffeic, syringic, p-coumaric acid, ferulic, Di-Caffeic) were quantified on the chlorogenic acid calibration curve (y = 22.585 − 36.728, r^2^ = 0.9937) with a minimum and maximum concentration of 10 to 50 μg/mL chlorogenic acid and expressed as chlorogenic equivalents (µg chlorogenic acid/g_dry weight_). All the analyses were made in triplicate and are presented as means ± standard deviations. The samples limit of quantification (LOQ) was 1 μg/mL and the limit of detection (LOD) was 0.125 μg/mL.

#### 2.5.9. Carotenoids Analysis

An Agilent 1200 HPLC system coupled to a diode array detector (Agilent-Technologies, Santa Clara, CA, USA), was used for the identification of carotenoids, as described by Szabo et al. [31]. Reversed-phase EC 250/4.6 Nucleodur 300–5 C-18 ec. column (250 × 4.6 mm), 5 μm (Macherey-Nagel, Düren, Germany) was used for carotenoid separation. The mobile phases were acetonitrile:water (9:1, *v*/*v*) with 0.25% triethylamine (A) and ethyl acetate with 0.25% triethylamine (B) with the elution program detailed described by Szabo et al. [31]. The flow rate was 1 mL/min, and the chromatograms were recorded at λ = 450 nm. A β-Carotene calibration curve from Sigma-Aldrich (Steinheum, Germany) was used for the quantification of individual carotenoids (y = 86.781x − 19.028, r^2^ = 0.9931), with a minimum and a maximum concentration of 1 µg/mL and 25 µg/mL, respectively. The analysis was carried out in triplicate and presented as means ± standard deviations.

#### 2.5.10. Ascorbic (AA) and Dehydroascorbic (DHAA) Acids Determination through HPLC-DAD-ESI-MS

Ascorbic and dehydroascorbic acids were performed on an HPLC-DAD-ESI-MS system, composed of an Agilent 1200 HPLC equipped with a quaternary pump, autosampler, DAD detector, coupled to an MS-detector single-quadrupole Agilent 6110 (Agilent-Technologies, Santa Clara, CA, USA). The compounds separation was made on an XDB C18 Eclipse column (4.5 × 150 mm, particle size 5 μm) with the following binary gradients: 1% formic acid:acetonitrile (95:5) in distilled water (*v*/*v*) (A) and 1% formic acid in acetonitrile (B); the flow rate was 0.5 mL/min at a temperature of 25 ± 0.5 °C. A scanning range of 100–600 *m/z* in the ESI (+) mode was performed for the MS fragmentation and the capillary voltage was set at 3000 V, temperature 300 °C, with a nitrogen flow of 7 L/min. The spectral absorbance values were registered in the range 200–400 nm and the chromatograms were recorded at λ 240 nm. To acquire and analyze the samples, an Agilent ChemStation software (Rev B.04.02 SP1, Palo Alto, CA, USA) was used. Acid ascorbic standard was used for identification and quantification (y = 95.421x − 391.07, r^2^ = 0.0059). All samples were analyzed in triplicate and expressed as means ± standard deviations.

#### 2.5.11. Folate Determination through HPLC-DAD-ESI-MS Assay

The same HPLC-DAD-ESI-MS system described above was used for the folate determination. Briefly, the separation was performed on the XDB C18 Eclipse column (4.5 × 150 mm, particle size 5 μm) with a mobile phase with acetonitrile:acetic acid 1% at a ratio of 20:80 (*v*/*v*) in the isocratic system, a flow rate of 0.5 mL/min, and temperature of 25 ± 0.5 °C. A scanning range of 120–600 *m/z* in the ESI (+) mode was applied for the MS fragmentation and the following parameters were set for the capillary voltage: 3000 V, temperature 350 °C, and nitrogen flow at 7 L/min. Chromatograms were registered at wavelength λ = 280 nm and data acquisition was done by using Agilent ChemStation software (Rev B.04.02 SP1) as described in our previous work [30,33]. A folic standard curve (y = 126.25x − 16.283, r^2^ = 0.9945) with a minimum and maximum concentration of 1 µg/mL to 30 µg/mL was used. All the samples were analyzed in triplicate and the results were expressed as means ± standard deviations.

#### 2.5.12. Antioxidant Capacity (AC)

AC was determined using the free radical scavenging activity with the stable radical 2,2-diphenyl-1-picryl-hydrazyl-hydrate (DPPH) following Agudelo et al. [33] methodology in triplicate. Absorbance was measured at 515 nm with a UV-visible spectrophotometer (Helios Zeta, Thermo Electron Corporation, Loughborough, UK. The results were expressed as milligram Trolox equivalents (TE) per 100 g (mg TE/100 g).

#### 2.5.13. Minerals Determination

Sample mineralization was conducted according to Mihăiescu et al. [34]. Briefly, 0.5 g of sample was homogenized with HNO_3_ 65% and 3 mL H_2_O_2_, and mineralized in a Berghof MWS-2 (Berghof, Achalm, Germany), following the program parameters described by Mihăiescu et al. [34]. After, the samples were made up with ultrapure water to 25 mL volumetric flask. Furthermore, according to Senila et al. [35] the inductively coupled plasma optical emission spectrometry (ICP-OES) tool was used for sample analysis.

For analysis, s spectrometer Optima 5300DV (PerkinElmer, Waltham, MA, USA), with a dual viewing inductively coupled plasma optical emission coupled with CETAC 6000AT+ (CETAC, Omaha, NE, USA) ultrasonic nebulizer. The following parameters were used: 1300 W RF power, 15 L/min plasma flow, 2.0 L/min auxiliary flow, 0.8 L/min nebulizer flow, and the sample uptake rate was 1.5 mL/min. The delay time for washing between each sample and signal measurement was 180 s and high-purity argon was used to sustain plasma as a carrier gas. For calibration, multi-elemental solutions of 1000 mg/L ICP Standard Certipur^®^ (Merck, Darmstadt, Germany) were used.

### 2.6. Statistical Analysis

To evaluate the data analysis of mixtures and extrudates, an ANOVA (Statgraphics Centurion XVII software) test with a confidence level of 95% was used, (*p* < 0.05). The differences between means were evaluated through the Fisher test. Using Statgraphics Centurion XVII software (17.2.04 version, Statgraphics Technologies, Inc., The Plains, VA, USA) a high correlation analysis with a 95% significance level was applied between the extrusion parameters and textual characteristics of extrudates. Pearson correlation was used to better explain the relationship between antioxidant activity and other bioactive compounds. All samples were analyzed in triplicate and the final results were reported as means ± standard deviations.

## 3. Results & Discussions

### 3.1. Physicochemical Characteristics of Extrudates

Mean values of x_w_, a_w_, WAI, WSI, SWE, Hy, SEI, ρ_b_, and ε of extrudates are showed in Table 1. All rose hip enrichments studied provoked significant changes in x_w_, a_w_, WSI, Hy, and ρ_b_ with respect to control extruded (*p* < 0.05). When some of the rosehip powdered product was added x_w_, a_w_, and ρ_b_ decreased significantly (*p* < 0.05), therefore, there are more hygroscopic and more easily soluble. The control sample presented 5.62 (0.05) g_w_/100 g, like other studies of corn extrudates [21,30] and the rest of the extrudates ranged from 3.7 to 4.6 g_w_/100 g. Extrusion moisture loss in the control was 10%, like other studies [30]. However, extrusion moisture loss in enriched samples was 16–24%. Thus, in samples with rose hip powder incorporation, as there is an increase in fiber, there is a greater amount of water to be absorbed by this component and, consequently, greater will be the loss of water at the open of the die with the pressure difference. According to Karkle et al. [36] it seems that the vapor pressure inside the air, extensibility, and water-binding are strictly related to the moisture loss of the die. Furthermore, its emphasized the starch degree transformation, since the presence of ungelatinized starch decrease extensibility degree, meanwhile residual water is shut in the structure, rather than evaporating at vapor flashpoint. The values of a_w_ were similar to the other corn snacks obtained by García-Segovia et al. and Uribe-Wandurraga et al. [21,23]. The lowest a_w_ values were obtained in PPR4E and CDR4E.

WAI and WSI properties exhibit the interaction of extrudates with water. [37]. WAI shows the quantity of water absorbed by the extrudate when immersed in water [38], meanwhile, WSI could emphasize the molecular damage that can occur during extrusion due to the water solubilized components releasing [39]. As observed in Table 1, RMDR4E and RMDR8E showed the lowest WAI values, meanwhile, the WSI parameter registered the highest extended values Using MD, RMD, and CD for encapsulating rosehip significantly increased WSI values (*p* < 0.05). Furthermore, according to the soluble nature of these biopolymers, extrudates with MD, RMD, and CD presented water solubilized components that make them vulnerable to molecular damage. Samples with PP or without biopolymers presented WSI values lower than the other extruded samples. Thus, R4E, R8E, PPR4E, and PPR8E could be more stable samples. SWE mean values expressed as mL_swollen_/g_dry_ solid are also shown in Table 1. Extrudates with PP in the formulation significantly presented the highest values of SWE (*p* < 0.05), probably for the hydration of the protein structure. However, extrudates with CD in the formulation presented the lowest values; the control SWE was like other studies [23,30]. Adding rosehip powders in this study provoked an increase of Hy, most in RMDR4E.

Typical extrudate structures are due to the sudden expansion—at the exit—of the molten mass from the restricted die, from high pressure to atmospheric pressure [26]. Adding rosehip powders to extrusion mixtures increased the SEI values, except for R8E, which showed a slight SEI decreased than the control (Table 1). The significantly highest SEI values were found in MDR4E and RMDR4E. There was a significant Pearson correlation between SEI and WAI (−0.7385, *p* < 0.05). Other studies have reported the same link between these parameters [28,30]. High WSI values are related to molecular damage due to the ease of solubilization of a sample so, according to our results, samples with PP or without biopolymers presented WSI values lower than the other extruded samples therefore more stability.

Finally, Table 1 also includes ρ_b_ and ε of extrudates. Density is defined as a general extrudate property, that could indicate changes in material parameters such as cell structure, pores, and voids developed during the extrusion process; a porous structure is characterized by highly expanded extruded materials [40]. This porous structure was measured by ε. There were significant (*p* < 0.05) differences in ρ_b_ values between control and enriched samples, with the values of the control ρ_b_ being higher. ρ_b_ was related to x_w_ by significant Pearson correlation (0.7311, *p* < 0.05) as also shown by García-Segovia et al. [21]. ε values ranged from 90.8 to 93.1. This is a narrow range, and ε did not show a clear trend. The highest ε values were observed in R4E and RMDR4E; however, PPR8E, RMDR8E, and CDR4E also showed significant values higher than the control (*p* < 0.05). ρ_b_ and ε values of the control sample were like other corn extrudates [23,30].

Texture characteristics are included in Table 2. Texture is defined as one of the main characteristics of extruded snacks with a high influence on the final quality of food products [41]. The extruded snack products texture is characterized mainly by crunchiness and crispness [42]. W_c_ is described as the force applied on the first bite to break the sample [43] and could correlate to fracturability, as a sensory criterion [44].

Hardness and chewing are usually associated with F_p_ and F_s_ of extruded products. Hardness is defined as the necessary force to compress a solid substance between the molar teeth [40]. N_sr_ describes the number of fracture events during puncture [45] and N_0_ corresponds to the number of fractures throughout the test [44]. Adding of rosehip powders studied to mixtures to extrusion decreased W_c_ values except to RMDR4E. The significantly lowest W_c_ value was found in R8E. Samples with rosehip powders required less force in the first bite to break the extrudate. All extrudates with rosehip powders showed lower F_p_ values than control and lower F_s_ values too, except to RMDR4E which presented similar F_s_ than control. N_0_ was significantly higher in extrudates with rosehip powders in comparison with control. N_sr_ and N_0_ are associated with the crunchiness of extruded snacks [21]; in this study, the use of rosehip powders in mixtures to obtain snacks made that extrudates were crunchier. Authors mentioned that N_sr_ and N_0_ could be influenced by the pore size, and furthermore, could be related to the interface fractures propagation, according to Chanvrier et al. [46].

Correlation analysis was applied in order to better explain the relationship between texture parameters of extruded products and typical extrudate parameters; W_c_ was positively correlated with x_w_ (0.7175, *p* < 0.05) and F_p_ with x_w_ and ρ_b_ presenting values of Pearson correlation of 0.7870 (*p* < 0.05) and 0.6315 (*p* < 0.05), respectively. Other works with corn snacks also detected a significant positive correlation between F_p_ and ρ_b_ [30].

Mixtures and extrudates optical properties are showed in Table 3 (L*, a*, b*, C*, h*, ΔE_1_, and ΔE_2_). Both mixtures and extrudates with or without R did not show differences in the measurements taken on white and black backgrounds; therefore, they were not translucent and color coordinates CIE*L*a*b* and the values of chroma (C*) and tone (h*) were obtained directly from the equipment used for color measurement. This has also been observed in corn snacks previously [30]. In contrast, other studies [21,28] have shown differences in those measurements (white and black backgrounds) in corn extrudates. In the present study, rosehip powder addition had a negative influence on L* and h* parameters (*p* < 0.05) but a positive one regarding the a* value.

**Figure 1:**
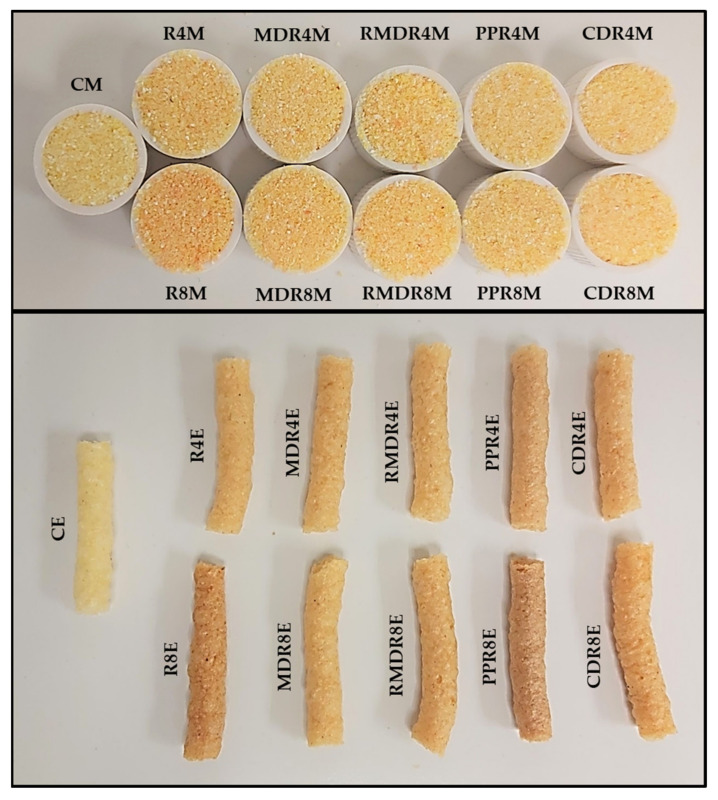
Mixtures (M) and extrudates (E) with different concentrations (4 and 8%) of rosehip preparation. R, rosehip; MDR, maltodextrin rosehip; RMDR, resistant maltodextrin rosehip; PPR, pea protein rosehip; CDR, cyclodextrin rosehip.

All mixtures were redder by the addition of rosehip or encapsulated rosehip. Moreover, these extrudates were also significantly (*p* < 0.05) redder than CE. R8M showed the highest a* and the lowest L*. The lowest values of b* and C* were in PPR4M and the highest values were in RMDR8M. After extrusion, L*, a* b*, and C* decreased significantly (*p* < 0.05). R8E presented the significantly (*p* < 0.05) highest values of a*, b*, and C*; however, the lowest h* was for PPR8. Moreover, CDR8E showed the significantly (*p* < 0.05) lowest value of L*. These differences can be observed in Figure 1.

Total color differences between samples with rosehip and the control (ΔE_1_) ranged between 4.4 and 13.5, higher than 3 units; therefore, humanly perceptible [47]. Color is an important quality parameter because it reflects the extent of chemical reactions and the degree of cooking or degradation that takes place during the extrusion process. ΔE_2_ represents the total color difference between extrudates and mixtures. According to Dogan et al. [48] extrusion provokes darker products with more intense yellow and red colors. In this study, ΔE_2_ ranged between 26.6 and 37.1. These ΔE_2_ values were significantly higher in CDR4E than in the other extrudates and lower in R8E compared to the rest.

Figure 1 shows the appearance of the mixtures and extrudates. In similarity with color coordinates, the reddish color (a* increase) of the mixture with increasing rose hip addition is remarkable. Moreover, extrudates lost the reddish color compared to mixtures, as observed in ΔE_2_. The appearance of the extrudates is adequate and in the same trend as these products.

### 3.2. Nutritional and Functional Value of Mixtures and Extrudates

#### 3.2.1. Phenolic Acid Profile of Mixtures and Extrudates 

The mixtures and extrudate phenolic acids content are presented in Table 4. The cornflour used in this study highlighted a total phenolic acid of 160.57 µg/g, from which the main amounts were represented by p-Coumaric, Di-caffeic, and ferulic acids with values 58.61_,_ 42.52, and 29.34 µg/g, respectively, whereas caffeic and syringic acids registered smaller values, 17.27 and 12.82 µg/g, respectively. The rosehip total phenolic amount was low compared with the corn flour and exhibited a value of only 27.09 µg/g, from which only ferulic and p-Coumaric acids were identified with values of 9.03 and 18.06 µg/g, respectively. The small amount of rosehip phenolic acids could be explained by rosehip being rich in chlorogenic acids and catechin, as previously shown by Tabaszewska et al. [7], acids not identified in this study.

**Figure 2:**
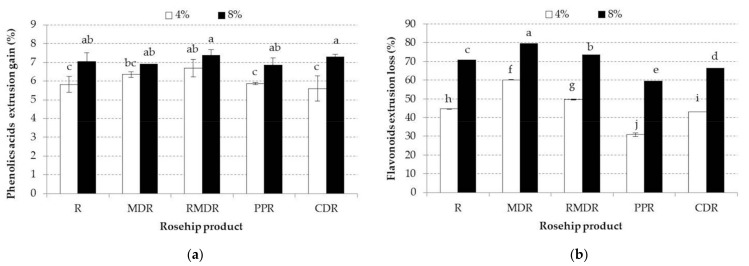
(**a**) Mean values and standard deviation of phenolic acids extrusion gain of samples enriched with different rosehip product at 4 and 8% concentration. (**b**) Mean values and standard deviation of phenolic acids extrusion loss of samples enriched with different rosehip products at 4 and 8% concentrations. Small different letters indicate significant changes between samples, by Fisher test (*p* < 0.05) for each analyzed parameter. R, rosehip; MDR, maltodextrin rosehip; RMDR, resistant maltodextrin rosehip; PPR, pea protein rosehip; CDR, cyclodextrin rosehip.

The phenolic acids from mixtures such as syringic acid and p-Coumaric acid were negatively influenced by the extrusion process (*p* < 0.05). However, in all samples with polymers, ferulic and Di-caffeic acids significantly increased their values through extrusion (*p* < 0.05) compared to the mixtures (Table 4 and Supplementary Tables S1 and S2). Total phenolic acids increase is shown in Figure 2a. Briefly, the total phenolic acids increase in extrusions ranged from 5.60% to 7.37% for CDR4E to RMDR8M samples, respectively.

The extrusion effect on the phenolic content is still controversial and represents an open field to be further exploited. For instance, there are several studies that showed a negative correlation between the extrusion process and bioactive compounds. Pasqualone et al. [4] highlighted the extrusion could have a negative effect on the different groups of phenolic compounds. Anton et al. [45] showed that navy and red beans are rich sources of antioxidant activity and phenolic compounds; however, through extrusion, their amounts decreased by 10% and 17% for navy beans and 70% and 62% for red beans, respectively. The authors explained the decrease of bioactive compounds was due to extrusion temperature, moisture of the extrusion material, and a possible polymerization between phenolic acids and tannin, which could involve a decrease in the compounds’ extractability, also reducing their antioxidant activities. In contrast, Arribas et al. [49] demonstrated that during an extrusion process, the phenolic groups are not affected to the same extend, highlighting that novel gluten-free expanded products based on pea flours increased their total phenolic content through extrusion.

Regarding flavonoids, one hydroxybenzoic acid (Di-Gallic acid) and ten flavonols were identified, as presented in Table 5. Adding rose hip increased the mixture's flavonoids amount, probably due to its rich flavonoid chemical composition, as previously shown [7,50].

During extrusion, flavonols like Quercetin-acetyl-rhamnoside, Isorhamnetin-glucoside, and Procyanidin dimmer were lost, as presented in Figure 2b. PPR4E and PPR8E samples registered the smallest extrusion losses, being statistically different from the other samples (*p* < 0.05). For instance, the extrudates with 4% rosehip registered a 60% loss for the MDR4M sample, whereas PPR4E registered a loss of 39% (Figure 2b). Regarding the MDR8M sample, the total flavonoids content was reduced by 80%, whereas PPR8E registered a loss of 63%. The results showed that during extrusion, PP highlighted the biggest flavonols protection, leading to minimal flavonols losses. The flavonoids decreasing amount during extrusion was also highlighted by Patil et al. [51] who showed that extrusion significantly reduced flavonoids in millet and sorghum flour.

#### 3.2.2. Carotenoids Profile of Mixtures and Extrudates

The increase of R4E and 8RE percentages increased the lutein, zeaxanthin, lycopene, β Carotene, Zea-ester, and Lut-ester contents; again highlighting rose hip’s substantial total carotenoid content. In this study, the rosehip showed a rich chemical composition of carotenoids, registering a total value of 208.35 µg/g. The largest amounts were represented by lycopene, β Carotene, Zea-ester, and Lut-ester as 78.95, 58.25, 44.54, and 14.32 µg/g, respectively. The high level of carotenoids content in the rosehip was also mentioned by Al-Yafeai et al. [52] and Kazaz et al. [53].

**Figure 3:**
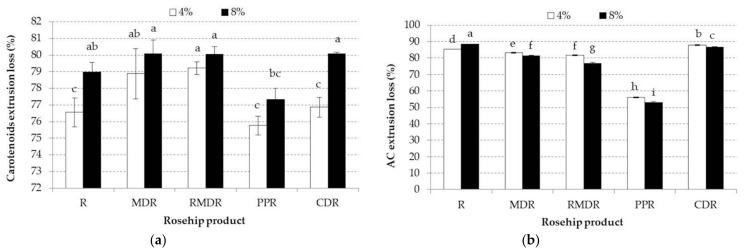
(**a**) Mean values and standard deviation of carotenoids extrusion loss of samples enriched with different rosehip products at 4 and 8% concentrations. (**b**) Mean values and standard deviation of antioxidant capacity (AC) extrusion loss of samples enriched with different rosehip products at 4 and 8% concentrations. Small different letters indicate significant changes between samples, by Fisher test (*p* < 0.05) for each analyzed parameter. R, rosehip; MDR, maltodextrin rosehip; RMDR, resistant maltodextrin rosehip; PPR, pea protein rosehip; CDR, cyclodextrin rosehip.

Adding rose hip increased the mixture’s content, leading to significant differences between samples with 4% and 8% addition, as presented in Table 5. Furthermore, Table 5 shows that the extrusion process leads to significant differences between mixtures and extrudates (*p* < 0.05). The carotenoids extrusion losses are illustrated in Figure 3a and show that samples with PPR8 registered the smallest carotenoids loss (77.2%), whereas, samples with MDR, RMDR, and CDR registered similar high losses (80%).

#### 3.2.3. Ascorbic and Dehydroascorbic Acids, Vitamin C, Folate, and Antioxidant Activity

Adding rosehip in different percentages in mixtures had a positive influence on AA and DHAA, vitamin C, folate content, and AC, as presented in Table 6. In this study, the amount of AA, DHAA, vitamin C, folate, and AC in rosehip were 3.83, 1.39, 5.22, and 306 µg/g, as well as 19.23 µgTE/g, respectively. Roman et al. [5] showed values between 112.20 mg/100 g and 360.22 mg/100 g for the AA concentration in frozen rosehip pulp and explained the difference due to several factors such as altitude variations, harvesting period, ecological factors, and species. Furthermore, Mihaylova et al. [54] reported the AA content could be influenced by the harvesting time, the method of extraction, and the solvent:plant material ratio. Moreover, Georgieva et al. [55] reported a significantly larger amount of vitamin C such as 110 mg/100 g.

The AC mixtures values increased with rosehip addition, mainly because of its high antioxidant activity (19.23 μg TE/g). A large body of literature emphasized the AC rosehip high content [7,50,55], and justify it mainly due to the presence of flavonoids, according to Selahvarzian et al. [50]. Moreover, carotenoids compounds have been also associated with antioxidant activity, due to their radical scavenging properties of singlet molecular oxygen and peroxyl radicals, being considered efficient ROS scavengers [56]. Carotenoids were mentioned to be involved also in the reduction of the membrane structures oxidation and therefore, on the morbidity risk [51].

**Figure 4:**
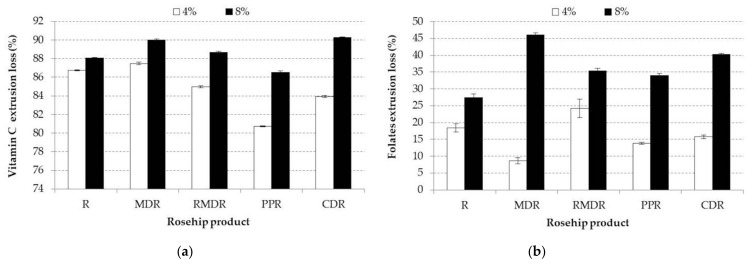
(**a**) Mean values and standard deviation of vitamin C extrusion loss of samples enriched with different rosehip products at 4 and 8% concentration. (**b**) Mean values and standard deviation of folates extrusion loss of samples enriched with different rosehip products 4 and 8% concentration. Small different letters indicate significant changes between samples, by Fisher test (*p* < 0.05) for each analyzed R, rosehip; MDR, maltodextrin rosehip; RMDR, resistant maltodextrin rosehip; PPR, pea protein rosehip; CDR, cyclodextrin rosehip.

AC extrudates and vitamin C values decrease during extrusion, even if polymers were used (Table 6, Figure 3b and Figure 4a). The reduction of AC during the extrusion process was mentioned also by Anton et al. and Arribas [45,49] who explained it through a possible polymerization between phenolic acids and tannins ending with a decrease of the compounds extractability, and therefore, on the antioxidant activity. Furthermore, extrusion temperature and moisture of the extrusion material could also be factors that might be involved in the AC reduction during the extrusion process [48]. This is in line with Potter et al. [56] who showed that during extrusion of fruit powders to obtain snacks, Maillard Reaction Products with antioxidant activity could be inhibited by fruit powders. The same authors reported that during the fruit extrusion process, a decrease in the antioxidant activity ranging from 15% to 50%, while the phenolic compound was not affected at all. This could be explained by the loss of antioxidants, other than the phenolic ones.

The samples folate content is presented in Table 6. The rosehip folate content (306 μg/g) influenced in a positive way the folate mixtures samples, being in concordance with Strålsjö et al. [57], who mentioned for rosehip a total value content ranging between 400 and 600 μg/g folate based on dry matter. With respect to folate extrudates, the highest values were registered by R8E (14.86 μg/g), meanwhile, the lowest values were recorded by CE (0.72 μg/g) and RMDR4E (5.81 μg/g) samples. The extrusion process led to a decrease in folate amount in all samples (Figure 4b). This could be explained by the key drivers involved in the folate amount decrease such as high temperature and low extrusion material moisture content, combined with screw speed, as mentioned by Gulati et al. [58].

The mixtures levels of AA and DHAA registered a linear increase with the addition of rosehip (Table 6), reaching the highest values for R8M and MDR8M samples, meanwhile vitamin C content exhibited the highest values for the same samples: 997.13 μg/g and 807.59 μg/g, respectively. The AA, DHAA, and vitamin C extruded contents were not protected against the extrusion process by the used biopolymers, probably due to the disintegration of wall material’s crystalline structure during the extrusion process [13]. The decrease of vitamin C during extrusion was also mentioned by Gulatti et al. [58] and Singh et al. [59], who stated that vitamin C is sensitive to heat and oxidation.

To better explain the relationship between vitamin C, AA, folates, total phenolic acids, total flavonoids, and total carotenoids, correlation statistical analyses were used. Positive strong Pearson’s correlation coefficients (0.9929, 0.9927, and 0.9907 (*p* < 0.05)) were identified between folates, AA, vitamin C and AC mixtures and extrudates. Total flavonoids and total carotenoids also showed high positive Pearson’s correlations of 0.9870 and 0.9615 (*p* < 0.05), respectively. Some authors have reported that the main contributing AC factor was vitamin C or AA [60,61]. However, other studies observed a high correlation between flavonoids and AC in grapefruit powders [62]. Likewise, Igual et al. [59] and Arilla et al. [63] found a significant correlation between the AA and TC content, like in the present study (0.9846, *p* < 0.05), probably because of the stabilizing effect of AA on carotenoids [64].

The nutrition labeling for foodstuffs from the Council [65] that the recommended daily allowance of folate is 200 μg. In line with this and regulation no. 1924/2006 of the European Parliament and of the Council of 20 December 2006 on nutrition and health claims made in foods [66], extrudates enrichment with rosehip are a food “high in folate.” Moreover, the intake of 14 g of R8E or 36 g of RMDR4E (extrudates with the highest and the lowest folates content, respectively) can give the recommended daily allowances of folate. Likewise, in the case of vitamin C, the recommended daily allowance is 60 mg, so only R8E is a “source of vitamin C.”

#### 3.2.4. Minerals Mixtures and Extrudates Content

In this study, the biggest amounts of rosehip macrominerals were registered by Ca (Calcium), K (Potassium), and Mg (Magnesium) with 7367.09, 6785.77, and 1558.9 µg/g values, meanwhile the micromineral values for Mn (Manganese), Fe (Iron) and Zn (Zinc) were 127.56, 98.36, and 20.56 µg/g, respectively.

According to the scientific literature, rosehip represents a rich source of minerals, such as K, Ca, Mg, Na, Mn, Fe, and Zn, as reported by a large body of literature [53,54,67,68]. For instance, Kazaz et al. [53] reported values of 1652.0 mg/kg for Na, 14,545.0 mg/kg for K, 8442.0 mg/kg for Ca, and 117.5 mg/kg for Fe. Furthermore, Mihaylova et al. [54] reported 73 mg/kg for Na, 7222 mg/kg for K, 3297 mg/kg Ca and 22 mg/kg for Fe, respectively. The differences between mineral composition could be influenced by harvest time, altitude, fruit size, ecological factors, variety, species [53], storage, and manipulation. 

Adding rose hip increased the mineral content in the mixtures (Table 7), probably because rosehip contains high amounts of minerals. Furthermore, the extrusion process also increased the mineral content (Table 7). The highest extrudate macromineral content was reached by sample PPR8E, registering values of 572.25, 637.40, 1518.03, and 257.61 µg/g for Na, Mg, K, and Ca, respectively, and registered values of 23.02, 13.93, and 10.14 µg/g for microminerals Fe, Mn, and Zn, respectively (Table 8). This could be explained by the fiber (crude fiber maximum of 10%, according to the product specification sheet) and phenolic compounds of pea protein powder; and also by its initial ash content (maximum of 10% according to the product specification sheet).

Gulati et al. [58] showed that mineral bioavailability could be influenced by the raw material dietary fibers and phenolic compounds. For instance, phenolic compounds anionic groups can bind essential mineral elements and during an extrusion process are degraded or polymerized, leading to their decrease chelating properties. Furthermore, it seems that extrusion processes can influence the cell wall polysaccharides content, leading to higher amounts of soluble and insoluble fibers which improved mineral absorption. Botelho et al. [69] reported that rosehip could also be a source of condensed and hydrolyzable tannins, from which oligomeric procyanidins were identified by Guimarães et al. [70], and ellagitannins reported by Fecka et al. [71]. Fetni et al. [72] also reported that rosehip methanol extract could have moderate levels of tannins. According to Rauf et al. [73], tannins could bind the mineral nutrient and therefore interfere in the digestion and absorption processes.

Kamau et al. [74] reported the extrusion parameters such as temperature, extruder screw speeds, and extrusion material moisture could reduce the tannin content by 98%. The strongest positive effect on different minerals, such as Fe and Cu, was obtained using extrusion [75], leading to higher amounts. In white lupine, Fe (4.10 mg/100 g dry weight) reached a value of 8.82 mg/100 g dry weight through extrusion, doubling its amount. Singh et al. [59] explained the enriched amounts of minerals through extrusion due to the reduction of antinutritional factors, such as condensed tannins and phytates. Moreover, the authors mentioned the chemical alteration of fiber could improve mineral absorption.

## 4. Conclusions

The addition of rosehip (*R. canina*) powder led to significant changes in physicochemical characteristics of extrudates compared to a control sample. xw, aw, and ρ_b_ showed significant decreases (*p* < 0.05) in all samples, whereas WSI and Hy increased significantly (*p* < 0.05). Extrudates with 4 or 8% rosehip encapsulated with pea protein registered the lowest WSI values, showing more sample stability. However, mixtures’ optical properties, such as reddish color a* increased with increasing rose hip content, but in line with the color of these types of products. Nutritionally, PPR8E registered the highest content of AC, vitamin C, and total flavonoids content such as Quercetin-glucoside, Quercetin-glucosyl-glucosyl-rhamnoside, Isorhamnetin-glucoside, and Isorhamnetin-acetyl-glucosyl-glucoside. PPR8E, R8E, and CDR8E exhibited the highest values of total carotenoid content, and macrominerals were identified in larger amounts in PPR8E, RMDR8E, and CDR8E.

To conclude, we can assess that the addition of 8% *Rosa canina* encapsulated with pea protein biopolymers could be successfully used in corn extrudates manufacturing.

## Figures and Tables

**Table 1:** Mean values (and standard deviations) of water content (x_w_), water activity (a_w_), water absorption index (WAI), water solubility index (WSI), swelling index (SWE), hygroscopicity (Hy), expansion index (SEI), bulk density (ρ_b_) and porosity (ε) of extrudates.

Sample	x_w_ (g_w_/100 g)	a_w_	WAI	WSI (%)	SWE (mL_swollen_/g_dry solid_)	Hy (g_w_/100 g_dry solid_)	SEI	ρ_b_ (g/cm^3^)	ε (%)
CE	5.62 (0.05) ^a^	0.327 (0.003) ^a^	4.135 (0.015) ^b^	16.81 (0.12) ^g^	2.279 (0.102) ^e^	21.69 (0.19) ^f^	13.1 (0.4) ^f^	0.103 (0.003) ^a^	91.3 (0.2) ^cd^
R4E	4.35 (0.05) ^bcd^	0.310 (0.003) ^b^	3.9 (0.2) ^cd^	20.3 (0.9) ^de^	2.59 (0.04) ^cd^	23.8 (0.3) ^bc^	13.9 (0.4) ^d^	0.078 (0.004) ^d^	93.0 (0.4) ^a^
MDR4E	4.49 (0.03) ^bc^	0.303 (0.003) ^c^	3.74 (0.02) ^def^	23.82 (0.13) ^b^	2.407 (0.012) ^cde^	24.1 (0.2) ^b^	15.4 (0.5) ^a^	0.090 (0.005) ^bc^	91.7 (0.5) ^bcd^
RMDR4E	4.565 (0.005) ^b^	0.303 (0.003) ^c^	3.246 (0.015) ^h^	26.21 (0.13) ^a^	2.576 (0.004) ^cd^	25.1 (0.2) ^a^	15.6 (0.4) ^a^	0.0795 (0.0004) ^bcd^	93.074 (0.009) ^a^
PPR4E	4.1 (0.2) ^de^	0.259 (0.003) ^h^	3.630 (0.012) ^efg^	20.95 (0.12) ^d^	4.7 (0.3) ^b^	22.71 (0.14) ^de^	14.6 (0.6) ^c^	0.081 (0.006) ^bcd^	91.9 (0.5) ^bc^
CDR4E	4.10 (0.03) ^de^	0.258 (0.003) ^h^	3.775 (0.006) ^cde^	23.32 (0.02) ^b^	1.70 (0.17) ^fg^	22.3 (0.7) ^ef^	15.1 (0.4) ^b^	0.090 (0.009) ^b^	92.3 (0.6) ^ab^
R8E	4.0 (0.3) ^e^	0.274 (0.003) ^g^	4.48 (0.02) ^a^	18.51 (0.17) ^f^	2.674 (0.012) ^c^	23.5 (0.6) ^bc^	12.4 (0.4) ^g^	0.084 (0.002) ^bcd^	90.8 (0.3) ^d^
MDR8E	4.16 (0.02) ^de^	0.299 (0.003) ^d^	4.12 (0.07) ^b^	21.65 (0.07) ^c^	1.98 (0.05) ^f^	23.72 (0.05) ^bc^	13.9 (0.4) ^d^	0.079 (0.006) ^bcd^	92.0 (0.7) ^bc^
RMDR8E	4.34 (0.06) ^bcd^	0.300 (0.003) ^cd^	3.56 (0.03) ^g^	25.8 (0.2) ^a^	2.34 (0.14) ^de^	22.41 (0.08) ^e^	14.1 (0.3) ^d^	0.079 (0.009) ^cd^	92.3 (0.2) ^ab^
PPR8E	3.70 (0.09) ^f^	0.278 (0.003) ^f^	3.882 (0.003) ^c^	19.5 (0.4) ^e^	5.68 (0.17) ^a^	23.2 (0.2) ^cd^	14.2 (0.3) ^d^	0.075 (0.004) ^d^	92.6 (0.4) ^ab^
CDR8E	4.26 (0.05) ^cde^	0.290 (0.003) ^e^	3.61 (0.05) ^fg^	24.0 (0.3) ^b^	1.681 (0.108) ^g^	23.37 (0.03) ^cd^	13.5 (0.2) ^e^	0.0861 (0.0009) ^bcd^	92.02 (0.12) ^bc^

Values not sharing the same small letter in a column indicate significant changes between samples, by Fisher test (*p* < 0.05). Samples were extrudates (E), with different concentrations (4 and 8%) of rosehip preparation. R, rosehip; MDR, maltodextrin rosehip; RMDR, resistant maltodextrin rosehip; PPR, pea protein rosehip; CDR, cyclodextrin rosehip; x_w_: water content a_w_: water activity, WAI: water absorption index WSI: water solubility index SWE: swelling index, Hy: hygroscopicity, SEI: expansion index, ρ_b_: bulk density, ε: porosity.

**Table 2:** W_c_, N_sr_, F_s_, F_p,_ and N_0_ extrudates mean values (and standard deviations).

Sample	W_c_ (N × mm)	N_sr_ (mm^−1^)	F_s_ (N)	F_p_ (N)	N_0_
CE	0.20 (0.03) ^a^	10.1 (0.9) ^bc^	2.1 (0.3) ^a^	1.6 (0.2) ^a^	106 (8) ^d^
R4E	0.12 (0.03) ^bc^	11.1 (1.6) ^bc^	1.3 (0.3) ^bcd^	1.0 (0.2) ^bc^	123 (14) ^bcd^
MDR4E	0.13 (0.04) ^b^	11.5 (1.9) ^b^	1.4 (0.2) ^b^	1.0 (0.3) ^b^	126 (21) ^bc^
RMDR4E	0.21 (0.05) ^a^	10.7 (1.2) ^bc^	2.5 (0.5) ^a^	1.1 (0.2) ^b^	127 (14) ^bc^
PPR4E	0.099 (0.013) ^cd^	11.61 (1.02) ^b^	1.4 (0.3) ^b^	0.9 (0.2) ^bc^	137 (7) ^ab^
CDR4E	0.110 (0.013) ^bc^	10.0 (0.8) ^c^	1.09 (0.08) ^cde^	0.77 (0.06) ^cd^	115 (5) ^cd^
R8E	0.060 (0.004) ^e^	10.8 (1.4) ^bc^	0.728 (0.102) ^f^	0.48 (0.05) ^e^	121 (14) ^bcd^
MDR8E	0.097 (0.016) ^cd^	14.0 (1.2) ^a^	1.34 (0.19) ^bc^	1.1 (0.2) ^bc^	145 (14) ^a^
RMDR8E	0.095 (0.017) ^cd^	11.04 (1.02) ^bc^	1.05 (0.19) ^de^	0.73 (0.17) ^d^	125 (8) ^bc^
PPR8E	0.075 (0.007) ^de^	13.12 (0.12) ^a^	1.04 (0.04) ^de^	0.77 (0.09) ^cd^	134 (15) ^ab^
CDR8E	0.078 (0.003) ^de^	11.0 (1.2) ^bc^	0.85 (0.06) ^ef^	0.62 (0.13) ^de^	127(19) ^bc^

Values not sharing the same small superscript letter in a column indicate significant changes between samples, by Fisher test (*p* < 0.05). Samples were extrudates (E), with different concentrations (4 and 8%) of rosehip preparation. R, rosehip; MDR, maltodextrin rosehip; RMDR, resistant maltodextrin rosehip; PPR, pea protein rosehip; CDR, cyclodextrin rosehip; crispness work (Wc), the spatial frequency of structural ruptures (Nsr), the average specific force of structural ruptures (Fs), average puncturing force (Fp), and several peaks (N_0_) of extrudates.

**Table 3:** Color coordinates (L*, a*, b*, C*, and h*) and total color differences (ΔE) of corn mixtures and extrudates.

Parameter	L*	a*	b*	C	H	ΔE_1_	ΔE_2_
	**Mixtures**						
CM	79.9 (0.6) ^aA^	6.4 (0.5) ^gA^	41 (3) ^abcA^	42 (3) ^cA^	81.1 (0.3) ^aB^	-	-
R4M	74.4 (0.2) ^dA^	13.4 (0.6) ^cA^	40.4 (0.5) ^bcA^	42.6 (0.3) ^bcA^	71.59 (1.02) ^gB^	8.9 (0.7) ^cA^	-
MDR4M	76.7 (0.2) ^cA^	11.0 (0.2) ^eA^	41.0 (1.6) ^bcA^	42.4 (1.6) ^cA^	75.0 (0.3) ^cdA^	5.7 (0.2) ^dB^	-
RMDR4M	76.3 (0.5) ^cA^	11.6 (0.3) ^deA^	41.2 (1.5) ^abcA^	42.8 (1.4) ^bcA^	74.3 (0.7) ^deB^	6.4 (0.4) ^dA^	-
PPR4M	76.2 (0.2) ^cA^	9.7 (0.2) ^fA^	36.9 (0.8) ^dA^	38.1 (0.7) ^dA^	75.2 (0.5) ^cA^	6.5 (0.6) ^dB^	-
CDR4M	77.4 (0.4) ^bA^	9.6 (0.6) ^fA^	42.4 (1.4) ^abA^	43.5 (1.5) ^abcA^	77.2 (0.4) ^bA^	4.39 (1.09) ^eB^	-
R8M	71.0 (0.3) ^fA^	15.8 (0.8) ^aA^	40 (2) ^cA^	43 (2) ^abcA^	68.5 (0.8) ^hA^	13.0 (0.6) ^aA^	-
MDR8M	73.8 (0.5) ^eA^	14.2 (0.3) ^bB^	42.5 (1.7) ^abB^	44.8 (1.7) ^abB^	71.5 (0.4) ^gB^	10.1 (0.8) ^bA^	-
RMDR8M	74.5 (0.7) ^dA^	13.1 (0.6) ^cA^	43.3 (1.4) ^aA^	45.2 (1.5) ^aA^	73.1(0.6) ^fA^	9.0 (0.9) ^cA^	-
PPR8M	73.6 (0.8) ^eA^	10.2 (0.4) ^fA^	37.7 (0.9) ^dA^	39.06 (1.03) ^dA^	74.9 (0.3) ^cdA^	8.1 (0.4) ^cB^	-
CDR8M	76.6 (0.3) ^cA^	11.68 (0.08) ^dA^	40.75 (1.03) ^bcA^	42.4 (0.9) ^cA^	74.0 (0.4) ^eA^	6.3 (0.3) ^dB^	-
	**Extrudates**						
CE	56 (2) ^aB^	0.148 (0.013) ^gB^	17.9 (0.7) ^eB^	17.9 (0.7) ^eB^	89.53 (0.03) ^aA^	-	34 (2) ^abc^
R4E	52 (2) ^bcdB^	5.6 (0.6) ^deB^	21.0 (0.6) ^bcB^	21.7 (0.7) ^bcdB^	75.2 (1.2) ^cdA^	7.4 (1.8) ^deA^	30.75 (1.03) ^cd^
MDR4E	52 (3) ^bcB^	5.3 (0.7) ^efB^	20.8 (2.3) ^bcB^	21 (2) ^bcdB^	75.6 (0.9) ^bcA^	7.5 (1.5) ^deA^	32 (3) ^bcd^
RMDR4E	55 (2) ^abB^	4.7 (0.3) ^fB^	20.0 (1.8) ^bcdB^	20.6 (1.8) ^cdB^	76.7 (0.9) ^bA^	5.7 (0.5) ^eA^	31 (3) ^bcd^
PPR4E	52 (3) ^bcdB^	6.2 (0.5) ^cdB^	20.2 (1.7) ^bcdB^	21.2 (1.7) ^bcdB^	73.0 (0.5) ^efB^	8.2 (1.4) ^dA^	30 (4) ^de^
CDR4E	48.6 (1.4) ^deB^	5.2 (0.5) ^efB^	19.3 (0.6) ^cdeB^	20.0 (0.7) ^cdeB^	74.9 (1.3) ^cdB^	9.0 (1.5) ^cdA^	37.12 (1.08) ^a^
R8E	50.1 (0.9) ^cdeB^	10.3 (0.5) ^aB^	24.8 (0.5) ^aB^	26.8 (0.6) ^aB^	67.5 (0.6) ^gA^	13.5 (0.7) ^aA^	26.6 (0.8) ^e^
MDR8E	54.0 (1.5) ^abB^	6.3 (0.8) ^cdB^	22.0 (1.4) ^bB^	22.9 (1.6) ^bB^	74.04 (1.06) ^deA^	7.8 (1.6) ^deB^	29.5 (1.4) ^de^
RMDR8E	49 (2) ^deB^	6.8 (0.4) ^bcB^	21.07 (1.04) ^bcB^	22.13 (1.09) ^bcB^	72.2 (0.4) ^fA^	10.4 (1.3) ^bcA^	35 (2) ^ab^
PPR8E	49.8 (1.3) ^cdeB^	7.3 (0.7) ^bB^	18.3 (1.3) ^deB^	19.7 (1.4) ^deB^	68.3 (0.9) ^gB^	9.5 (0.8) ^bcdA^	30.9 (1.7) ^cd^
CDR8E	48 (4) ^eB^	6.6 (0.5) ^bcB^	20 (2) ^bcdB^	21 (2) ^bcdB^	71.7 (0.7) ^fB^	11.3 (2.8) ^bA^	36 (4) ^a^

Values not sharing the same small superscript letter in a row indicate significant changes between samples, by Fisher test (*p* < 0.05) comparing samples in mixtures or extrudates. Values not sharing the same capital letter in a column indicate significant changes between samples, by Fisher test (*p* < 0.05) comparing samples in mixtures or extrudates. Samples were mixtures (M) and extrudates (E), with different concentrations (4 and 8%) of rosehip preparation. R, rosehip; MDR, maltodextrin rosehip; RMDR, resistant maltodextrin rosehip; PPR, pea protein rosehip; CDR, cyclodextrin rosehip.

**Table 4:** Main phenolic acids (p-Courmatic acid, ferulic acid, Di-caf, expressed in µg/g_dry weight_) and flavonols content (Q-gluc, Q-glu-gluc-rham, I-gluc, I-acet-gluc-gluc, expressed in µg/g_dry weight_) of corn mixtures and extrudates.

Samples	p-Coumaric Acid	Ferulic Acid	Di-caff	Q-gluc	Q-glu-gluc-rham	I-gluc	I-acet-gluc-gluc
**Mixtures**							
CM	64.75 (0.21) ^aA^	42.86 (0.04) ^aA^	58.61 (0.06) ^aA^	-^k^	-^h^	-^i^	-^j^
R4M	61.95 (0.05) ^bA^	31.30 (0.09) ^bB^	44.47 (0.03) ^bB^	18.29 (0.03) ^gA^	18.33 (0.08) ^dB^	19.96 (0.08) ^eB^	17.62 (0.12) ^gB^
MDR4M	61.56 (0.03) ^dA^	31.00 (0.12) ^dB^	44.22 (0.51) ^bB^	16.35 (0.07) ^jB^	18.00 (0.09) ^eB^	20.26 (0.50) ^eB^	14.93 (0.02) ^iB^
RMDR4M	61.21 (0.02) ^eA^	30.83 (0.22) ^eB^	44.09 (0.05) ^bB^	18.27 (0.04) ^gB^	16.67 (0.05) ^gB^	18.29 (0.45) ^gB^	18.90 (0.07) ^fA^
PPR4M	61.76 (0.06) ^cA^	31.13 (0.05) ^cB^	44.27 (0.03) ^bB^	19.23 (0.02) ^fB^	16.62 (0.02) ^gB^	14.66 (0.02) ^hB^	16.29 (0.34) ^hB^
CDR4M	61.54 (0.04) ^dA^	30.82 (0.34) ^eB^	44.09 (0.09) ^bB^	17.59 (0.05) ^iB^	17.59 (0.55) ^fB^	19.51 (0.34) ^fB^	17.93 (0.77) ^gB^
R8M	59.55 (0.13) ^fgA^	29.49 (0.13) ^fB^	42.93 (0.02) ^cdB^	44.40 (0.04) ^aA^	28.42 (0.08) ^bB^	32.22 (0.09) ^aB^	27.77 (0.08) ^aB^
MDR8M	59.56 (0.02) ^fgA^	29.22 (0.07) ^gB^	42.84 (0.08) ^cdB^	39.83 (0.03) ^bA^	31.67 (0.04) ^aA^	29.60 (0.11) ^cA^	26.75 (0.55) ^bB^
RMDR8M	59.40 (0.15) ^ghA^	29.03 (0.08) ^hB^	42.74 (0.02) ^dB^	37.83 (0.08) ^cA^	28.41 (0.09) ^bB^	29.60 (0.05) ^cB^	25.80 (0.09) ^dB^
PPR8M	59.38 (0.09) ^hA^	29.05 (0.23) ^hB^	43.34 (0.84) ^cB^	37.12 (0.09) ^dA^	31.59 (0.03) ^aB^	28.98 (0.03) ^dB^	26.39 (0.05) ^cB^
CDR8M	59.59 (0.03) ^fA^	28.99 (0.78) ^hB^	42.70 (0.15) ^dB^	29.98 (0.03) ^eB^	23.78 (0.02) ^cB^	30.31 (0.02) ^bA^	24.44 (0.03) ^eA^
**Extrudates**							
CE	60.36 (0.05) ^aB^	32.52 (0.23) ^dB^	46.79 (0.40) ^eB^	-^k^	-^j^	-^g^	-^j^
R4E	57.73 (0.13) ^bB^	37.92 (0.24) ^aA^	53.56 (0.12) ^aA^	17.02 (0.02) ^iB^	31.66 (0.07) ^eA^	29.13 (0.04) ^eA^	19.83 (0.03) ^gA^
MDR4E	57.75 (0.07) ^bB^	37.76 (0.08) ^abA^	53.31 (0.13) ^aA^	19.56 (0.05) ^hA^	27.11 (0.55) ^hA^	26.01 (0.06) ^fA^	16.12 (0.02) ^iA^
RMDR4E	57.76 (0.04) ^bB^	37.78 (0.08) ^abA^	53.26 (0.22) ^abA^	23.59 (0.23) ^gA^	28.27 (0.03) ^fgA^	27.97 (0.38) ^eA^	16.46 (0.04) ^iB^
PPR4E	57.48 (0.11) ^cB^	37.81 (0.07) ^aA^	53.31 (0.33) ^aA^	25.96 (0.34) ^eA^	36.80 (0.78) ^cA^	31.47 (0.78) ^dA^	23.97 (0.65) ^fA^
CDR4E	57.36 (0.15) ^dB^	37.60 (0.09) ^bA^	52.71 (0.66) ^bA^	28.54 (0.04) ^dA^	27.82 (0.03) ^ghA^	28.45 (0.56) ^eA^	18.85 (0.05) ^hA^
R8E	55.35 (0.04) ^fB^	36.62 (0.13) ^cA^	51.91 (0.07) ^cA^	33.09 (0.08) ^bB^	39.56 (0.22) ^bA^	40.05 (0.22) ^bA^	34.30 (0.67) ^bA^
MDR8E	55.37 (0.08) ^efB^	36.65 (0.22) ^cA^	51.63 (0.23) ^cdA^	25.35 (0.06) ^fB^	24.43 (0.19) ^iB^	29.09 (0.34) ^eA^	29.09 (0.34) ^eA^
RMDR8E	55.46 (0.07) ^eB^	36.73 (0.31) ^cA^	51.50 (0.22) ^cdA^	25.09 (0.45) ^fB^	34.76 (0.34) ^dA^	34.70 (0.05) ^cA^	34.70 (0.05) ^cA^
PPR8E	55.20 (0.15) ^gB^	36.54 (0.15) ^cA^	51.17 (0.07) ^dA^	36.92 (0.57) ^aA^	43.47 (0.67) ^aA^	45.55 (1.06) ^aA^	45.55 (1.06) ^aA^
CDR8E	55.41 (0.02) ^efB^	36.60 (0.06) ^cA^	51.43 (0.02) ^cdA^	31.60 (0.03) ^cA^	28.81 (0.94) ^fA^	25.38 (0.02) ^fB^	25.38 (0.02) ^fB^

Small different letters in superscript within column indicates significant changes between samples, by Fisher test (*p* < 0.05) comparing studied samples in mixtures or extrudates. Big different letter within column indicates significant changes between samples, by Fisher test (*p* < 0.05) comparing mixtures and extrudates. R, rosehip; MDR, maltodextrin rosehip; RMDR, resistant maltodextrin rosehip; PPR, pea protein rosehip; CDR, cyclodextrin rosehip. 4, the concentration of 4% of rosehip preparation; 8, the concentration of 8% of rosehip preparation. M, mixture; E, extrudate. Di-caff: Di-caffeic acid; Q-gluc: Quercetin-glucoside; Q-glu-gluc-rham: Quercetin-glucosyl-glucosyl-rhamnoside; I-gluc: Isorhamnetin-glucoside; I-acet-gluc-gluc: Isorhamnetin-acetyl-glucosyl.

**Table 5:** Mean values (and standard deviations) of carotenoids content (µg/g_dry weight_) of corn mixtures and extrudates.

Samples	Lutein	Zeaxanthin	Lycopene	β Carotene	Zea-ester	Lut-ester	Total Carotenoids
	**Mixtures**						
CM	1.52 (0.02) ^fA^	3.52 (0.07) ^dA^	0.43 (0.02) ^fA^	0.47 (0.09) ^hA^	0.29 (0.06) ^gA^	0.26 (0.02) ^cA^	6.47 (0.28) ^gA^
R4M	1.86 (0.13) ^dA^	4.17 (0.19) ^cA^	8.23(0.04) ^dA^	5.03 (0.02) ^dA^	4.16 (0.32) ^eA^	1.28 (0.03) ^bA^	24.73 (0.73) ^dA^
MDR4M	1.79 (0.15) ^deA^	3.99 (0.11) ^cA^	7.86 (0.03) ^dA^	4.62 (0.23) ^efA^	4.03 (0.15) ^efA^	1.05 (0.10) ^bA^	23.34 (0.77) ^eA^
RMDR4M	1.67 (0.19) ^deA^	4.03 (0.02) ^cA^	7.83 (0.16) ^dA^	4.27 (0.17) ^gA^	3.91 (0.04) ^efA^	1.01 (0.08) ^bA^	22.73 (0.66) ^efA^
PPR4M	1.53 (0.25) ^fA^	4.07 (0.16) ^cA^	7.85 (0.19) ^dA^	4.86 (0.04) ^deA^	4.11 (0.23) ^efA^	1.13 (0.12) ^bA^	23.55 (0.99) ^eA^
CDR4M	1.77 (0.09) ^deA^	3.97 (0.44) ^cA^	7.13 (0.14) ^eA^	4.41 (0.03) ^fgA^	3.87 (0.06) ^fA^	1.00 (0.03) ^bA^	22.14 (0.79) ^fA^
R8M	2.81 (0.07) ^aA^	6.31 (0.33) ^aA^	17.30 (0.42) ^aA^	9.69 (0.06) ^aA^	8.03 (0.22) ^dA^	3.59 (0.37) ^aA^	47.73 (1.47) ^aA^
MDR8M	2.22 (0.18) ^cA^	5.73 (0.06) ^bA^	16.45 (0.09) ^bcA^	8.61 (0.26) ^bA^	8.6 (0.2) ^bA^	3.48 (0.21) ^aA^	44.29 (0.83) ^bA^
RMDR8M	2.24 (0.02) ^bcA^	5.58 (0.08) ^bA^	16.06 (0.06) ^cA^	8.10 (0.23) ^cA^	8.21 (0.11) ^cdA^	3.62 (0.16) ^aA^	43.07 (0.66) ^cA^
PPR8M	2.52 (0.03) ^bA^	6.29 (0.04) ^aA^	16.79 (0.40) ^bA^	9.53 (0.05) ^aA^	9.71 (0.07) ^aA^	3.68 (0.30) ^aA^	47.65 (0.89) ^aA^
CDR8M	2.36 (0.24) ^bcA^	5.62 (0.06) ^bA^	16.42 (0.03) ^bcA^	8.69 (0.07) ^bA^	8.50 (0.05) ^bcA^	3.55 (0.28) ^aA^	44.36 (0.73) ^bA^
	**Extrudates**						
CE	0.40 (0.03) ^fB^	0.60 (0.02) ^bB^	0.14 (0.02) ^fB^	0.17 (0.02) ^fB^	0.12 (0.03) ^hB^	0.13 (0.02) ^eB^	1.56 (0.14) ^fB^
R4E	0.60 (0.02) ^cdeB^	0.93 (0.23) ^bB^	1.76 (0.03) ^dB^	1.13 (0.08) ^dB^	0.89 (0.05) ^efB^	0.49 (0.03) ^cB^	5.80 (0.44) ^dB^
MDR4E	0.43 (0.06) ^fB^	0.82 (0.06) ^bB^	1.61 (0.05) ^eB^	0.98 (0.13) ^deB^	0.74 (0.08) ^fgB^	0.35 (0.08) ^ceB^	4.93 (0.46) ^eB^
RMDR4E	0.46 (0.03) ^fB^	0.73 (0.02) ^bB^	1.58 (0.04) ^eB^	0.89 (0.05) ^eB^	0.70 (0.30) ^gB^	0.37 (0.04) ^ceB^	4.72 (0.48) ^eB^
PPR4E	0.56 (0.07) ^eB^	0.89 (0.03) ^bB^	1.68 (0.07) ^deB^	0.99 (0.03) ^deB^	0.98 (0.02) ^deB^	0.60 (0.03) ^bcB^	5.70 (0.25) ^dB^
CDR4E	0.41 (0.02) ^fB^	0.86 (0.10) ^bB^	1.64 (0.09) ^deB^	0.86 (0.08) ^eB^	0.79 (0.04) ^fgB^	0.57 (0.04) ^bcB^	5.12 (0.37) ^eB^
R8E	0.81 (0.51) ^aB^	1.68 (0.09) ^aB^	3.25 (0.02) ^bB^	1.98 (0.15) ^aB^	1.50 (0.03) ^bB^	0.83 (0.15) ^abB^	10.04 (0.95) ^bB^
MDR8E	0.69 (0.04) ^bcB^	1.39 (0.49) ^aB^	3.13 (0.05) ^bcB^	1.76 (0.04) ^bB^	1.24 (0.07) ^cB^	0.78 (0.06) ^abB^	8.98 (0.75) ^cB^
RMDR8E	0.67 (0.02) ^cdB^	1.62 (0.13) ^aB^	3.04 (0.03) ^cB^	1.51 (0.16) ^cB^	1.11 (0.02) ^cdB^	0.79 (0.33) ^abB^	8.74 (0.69) ^cB^
PPR8E	0.77 (0.09) ^abB^	1.59 (0.03) ^aB^	3.66 (0.04) ^aB^	2.16 (0.04) ^aB^	1.81 (0.21) ^aB^	1.01 (0.07) ^aB^	11.00 (0.48) ^aB^
CDR8E	0.60 (0.03) ^deB^	1.34 (0.09) ^aB^	3.02 (0.16) ^cB^	1.68 (0.08) ^bcB^	1.46 (0.02) ^bB^	0.90 (0.13) ^aB^	8.99 (0.43) ^cB^

Small different letters in superscript within column indicates significant changes between samples, by Fisher test (*p* < 0.05) comparing studied samples in mixtures or extrudates. Big different letters within the column indicate significant changes between samples, by Fisher test (*p* < 0.05), comparing mixtures and extrudates. R, rosehip; MDR, maltodextrin rosehip; RMDR, resistant maltodextrin rosehip; PPR, pea protein rosehip; CDR, cyclodextrin rosehip. 4, the concentration of 4% of rosehip preparation; 8, the concentration of 8% of rosehip preparation. M, mixture; E, extrudates.

**Table 6:** Mean values (and standard deviations) of ascorbic acid (AA), dehydroascorbic acid (DHAA), vitamin C, folates, and antioxidant capacity. (AC) content (µg/gdry weight) of corn mixtures and extrudates.

Samples	AA	DHAA	Vitamin C	Folates	AC (TEq)
	**Mixtures**				
CM	77.72 (0.44) ^kA^	126.60 (0.37) ^kA^	204.33 (0.81) ^jA^	0.80 (0.03) ^iA^	98.6 (1.3) ^kA^
R4M	401.34 (0.07) ^eA^	267.77 (0.24) ^dA^	669.11 (0.31) ^dA^	11.40 (0.35) ^eA^	1480 (6) ^cA^
MDR4M	315.95 (0.57) ^gA^	254.91 (0.29) ^fA^	570.86 (0.85) ^fA^	7.59 (0.16) ^gA^	920 (3) ^fA^
RMDR4M	300.67 (0.27) ^hA^	238.94 (0.23) ^gA^	539.61 (0.50) ^gA^	7.68 (0.18) ^gA^	796 (2) ^hA^
PPR4M	195.71 (0.35) ^jA^	219.24 (0.33) ^iA^	414.95 (0.68) ^iA^	6.75 (0.29) ^hA^	326 (2) ^jA^
CDR4M	288.61 (0.39) ^iA^	207.73 (0.40) ^jA^	496.34 (0.80) ^hA^	8.49 (0.28) ^fA^	1209 (2) ^dA^
R8M	761.01 (0.54) ^aA^	236.12 (0.33) ^hA^	997.13 (0.88) ^aA^	20.47 (0.25) ^aA^	2350.1 (1.4) ^aA^
MDR8M	503.70 (0.66) ^cA^	303.88 (0.09) ^aA^	807.59 (0.75) ^bA^	18.66 (0.15) ^bA^	1054.4 (0.9) ^eA^
RMDR8M	441.54 (0.30) ^dA^	260.67 (0.04) ^eA^	702.21 (0.35) ^cA^	16.01 (0.50) ^cA^	873 (6) ^gA^
PPR8M	335.98 (0.21) ^fA^	272.00 (0.53) ^bA^	607.97 (0.74) ^eA^	14.45 (0.26) ^dA^	508 (3) ^iA^
CDR8M	537.142 (0.43) ^bA^	269.81 (0.30) ^cA^	806.95 (0.73) ^bA^	19.03 (0.36) ^bA^	1822 (3) ^bA^
	**Extrudates**				
CE	35.12 (0.41) ^hB^	23.10 (0.12) ^fB^	58.22 (0.52) ^hB^	0.72 (0.03) ^gA^	11.8 (1.3) ^hB^
R4E	55.50 (0.20) ^bB^	33.30 (0.23) ^aB^	88.80 (0.43) ^bB^	9.30 (0.14) ^dB^	216.8 (1.4) ^cB^
MDR4E	39.41 (0.51) ^gB^	32.03 (0.31) ^bB^	71.44 (0.83) ^gB^	6.94 (0.21) ^fA^	153 (3) ^fB^
RMDR4E	50.43 (0.31) ^dB^	30.52 (0.29) ^cdB^	80.95 (0.60) ^cdB^	5.81 (0.08) ^eB^	146.4 (1.4) ^gB^
PPR4E	47.13 (0.31) ^fB^	32.78 (0.53) ^aB^	79.91 (0.84) ^defB^	5.82 (0.27) ^eA^	144 (2) ^gB^
CDR4E	48.79 (0.30) ^eB^	30.95 (0.28) ^cB^	79.74 (0.58) ^defB^	7.15 (0.19) ^fB^	148 (3) ^gB^
R8E	85.50 (0.30) ^aB^	33.32 (0.15) ^aB^	118.82 (0.45) ^aB^	14.86 (0.41) ^aB^	270.9 (0.5) ^aB^
MDR8E	50.76 (0.34) ^dB^	29.87 (0.48) ^deB^	80.63 (0.82) ^cdeB^	10.06 (0.19) ^cB^	195.4 (0.9) ^eB^
RMDR8E	48.41 (0.22) ^eB^	31.16 (0.47) ^cB^	79.57 (0.68) ^efB^	10.35 (0.19) ^cB^	202 (2) ^dB^
PPR8E	51.79 (0.66) ^cB^	29.99 (0.21) ^deB^	81.79 (0.87) ^cB^	9.53 (0.10) ^dB^	238 (3) ^bB^
CDR8E	48.99 (0.17) ^eB^	29.60 (0.33) ^eB^	78.58 (0.50) ^fB^	12.36 (0.16) ^bB^	242 (4) ^bB^

Small different letters in superscript within column indicates significant changes between samples, by Fisher test (*p* < 0.05), comparing studied samples in mixtures or extrudates. Big different letters within the column indicate significant changes between samples, by Fisher test (*p* < 0.05), comparing mixtures and extrudates. R, rosehip; MDR, maltodextrin rosehip; RMDR, resistant maltodextrin rosehip; PPR, pea protein rosehip; CDR, cyclodextrin rosehip. 4, the concentration of 4% of rosehip preparation; 8, the concentration of 8% of rosehip preparation. M, mixture; E, extrudates.

**Table 7:** Macrominerals content (µg/g_dry weight_) of corn mixtures and extrudates.

Samples	Na	Mg	K	Ca	Total Macrominerals
**Mixtures**					
CM	41.65 (0.79) ^fB^	139.70 (0.93) _eB_	614.71 (0.88) ^fB^	61.26 (0.89) ^eB^	857.32 (3.49)
R4M	74.31 (0.89) ^cB^	240.02 (0.89) _cB_	964.11 (0.65) ^cB^	108.80 (0.77) ^cB^	1387.24 (3.20)
MDR4M	67.94 (0.80) ^deB^	190.44 (0.88) _dB_	884.90 (0.39) ^dB^	91.88 (0.48) ^dB^	1235.16 (2.55)
RMDR4M	65.55 (0.76) ^deB^	189.93 (0.57) _dB_	884.46 (0.78) ^dB^	90.59 (0.57) ^dB^	1230.53 (2.68)
PPR4M	65.62 (1.08) ^eB^	190.72 (0.24) _dB_	885.96 (0.81) ^dB^	91.61 (0.75) ^dB^	1233.91 (2.88)
CDR4M	68.31 (1.10) ^dB^	191.79 (0.89) _dB_	871.58 (0.96) ^eB^	91.96 (0.79) ^dB^	1223.64 (3.74)
R8M	96.79 (0.43) ^aB^	421.78 (0.77) _aB_	1399.21 (0.44) ^aB^	190.13 (0.65) ^aB^	2107.91 (2.29)
MDR8M	94.01 (0.27) ^bB^	402.15 (0.88) _bB_	1325.50 (0.77) ^bB^	176.01 (0.72) ^bB^	1997.67 (2.64)
RMDR8M	93.99 (0.93) ^bB^	400.00 (0.79) _bB_	1325.44 (0.28) ^bB^	175.10 (0.80) ^bB^	1994.53 (2.80)
PPR8M	93.67 (0.38) ^bB^	402.04 (0.97) _bB_	1326.10 (0.89) ^bB^	175.56 (0.19) ^bB^	1997.37 (2.43)
CDR8M	92.92 (0.25) ^bB^	400.55 (0.77) _bB_	1325.59 (0.85) ^bB^	175.22 (0.72) ^bB^	1994.28 (2.59)
**Extrudates**					
CE	90.17 (0.51) ^iA^	202.16 (0.73^) fA^	699.74 (0.84) ^hA^	119.60 (0.72) ^gA^	1111.67 (2.86)
R4E	339.08 (0.57) ^gA^	323.07 (0.49) ^dA^	1153.26 (0.73) ^fA^	209.67 (0.97) ^cA^	2025.08 (2.76)
MDR4E	353.50 (0.78) ^fA^	293.97 (0.89) ^eA^	984.21 (0.58) ^gA^	200.07 (0.25) ^dA^	1831.75 (2.50)
RMDR4E	394.11 (0.38) ^eA^	297.75 (0.88) ^eA^	984.93 (0.88) ^gA^	172.80 (0.78) ^fA^	1849.59 (2.92)
PPR4E	447.58 (0.93) ^cA^	328.11 (0.73) ^dA^	1159.71 (0.78) ^eA^	215.98 (0.39) ^cA^	2151.38 (2.83)
CDR4E	324.97 (0.69) ^hA^	289.06 (0.34) ^eA^	986.31 (0.59) ^gA^	189.99 (0.89) ^eA^	1790.33 (2.51)
R8E	393.57 (0.99) ^eA^	629.63 (0.77) ^aA^	1510.00 (0.82) ^bA^	251.35 (0.55) ^aA^	2784.55 (3.13)
MDR8E	412.54 (0.88) ^dA^	603.05 (0.99) ^bA^	1455.61 (0.69) ^cA^	239.05 (0.93) ^bA^	2710.25 (3.49)
RMDR8E	455.16 (1.09) ^bA^	591.40 (0.67) ^cA^	1451.61 (0.73) ^dA^	239.67 (0.64) ^bA^	2737.84 (3.13)
PPR8E	572.25 (0.88) ^aA^	637.40 (0.63) ^aA^	1518.03 (0.59) ^aA^	257.61 (0.72) ^aA^	2985.29 (2.82)
CDR8E	351.28 (0.79) ^fA^	590.17 (0.88) ^bcA^	1451.54 (0.64) ^dA^	239.11 (0.83) ^bA^	2632.10 (3.14)

Small different letters in superscript within column indicates significant changes between samples, by Fisher test (*p* < 0.05), comparing studied samples in mixtures or extrudates. Big different letters within a column indicate significant changes between samples, by Fisher test (*p* < 0.05), comparing mixtures and extrudates. R, rosehip; MDR, maltodextrin rosehip; RMDR, resistant maltodextrin rosehip; PPR, pea protein rosehip; CDR, cyclodextrin rosehip. 4, the concentration of 4% of rosehip preparation; 8, the concentration of 8% of rosehip preparation. M, mixture; E, extrudate.

**Table 8:** Microminerals content (µg/g_dry weight_) of corn mixtures and extrudates.

Samples	Cr	Mn	Fe	Ni	Cu	Zn	Se	Total Microminerals
**Mixtures**								
CM	0.25 (0.04) ^eB^	1.35 (0.29) ^fB^	14.58 (0.60) ^dB^	0.27 (0.03) ^eB^	0.86 (0.04) ^cB^	4.26 (0.12) ^deB^	0.60 (0.07) ^aA^	22.17 (1.19)
R4M	0.97 (0.03) ^cB^	5.95 (0.12) ^cB^	16.47 (0.55) ^cB^	0.54 (0.04) ^cB^	1.06 (0.10) ^bB^	5.98 (0.04) ^aB^	0.35 (0.02) ^bA^	31.31 (0.90)
MDR4M	0.76 (0.04) ^dB^	4.40 (0.21) ^deB^	13.99 (0.04) ^deB^	0.40 (0.07) ^deB^	0.82 (0.04) ^cB^	4.14 (0.03) ^eB^	0.23 (0.04) ^cdeA^	24.74 (0.47)
RMDR4M	0.79 (0.05) ^dB^	4.56 (0.06) ^dE^	14.10 (0.14) ^deB^	0.45 (0.03) ^cdB^	0.77 (0.06) ^cB^	4.36 (0.05) ^dB^	0.21 (0.03) ^defA^	25.17 (0.42)
PPR4M	0.69 (0.07) ^dB^	4.28 (0.08) ^deB^	13.92 (0.06) ^deB^	0.43 (0.04) ^cdB^	0.80 (0.03) ^cB^	4.25 (0.15) ^deB^	0.27 (0.02) ^cdA^	24.64 (0.45)
CDR4M	0.71 (0.04) ^dB^	4.17 (0.98) ^eB^	13.83 (0.08) ^eB^	0.42 (0.02) ^cdB^	0.83 (0.21) ^cB^	4.32 (0.03) ^deB^	0.30 (0.06) ^bcA^	24.58 (1.42)
R8M	1.51 (0.04) ^aB^	11.82 (0.13) ^aB^	19.68 (0.07) ^aB^	0.87 (0.06) ^aB^	1.35 (0.05) ^aB^	5.02 (0.20) ^bB^	0.17 (0.45) ^efgA^	40.22 (1.00)
MDR8M	1.22 (0.05) ^bB^	10.33 (0.16) ^bB^	17.39 (0.24) ^bB^	0.75 (0.11) ^abB^	1.23 (0.02) ^abB^	4.76 (0.07) ^cB^	0.14 (0.03) ^fgA^	36.36 (0.68)
RMDR8M	1.25 (0.03) ^bB^	10.20 (0.77) ^bB^	17.61 (0.38) ^bB^	0.77 (0.02) ^abB^	1.26 (0.51) ^aB^	4.67 (0.06) ^cB^	0.11 (0.02) ^gA^	35.87 (1.78)
PPR8M	1.19 (0.23) ^bB^	9.98 (0.03) ^bB^	17.31 (0.04) ^bB^	0.80 (0.03) ^abB^	1.23 (0.34) ^abB^	4.82 (0.04) ^cB^	0.17 (0.04) ^efgA^	35.50 (0.75)
CDR8M	1.11 (0.16) ^bB^	10.11 (0.33) ^bB^	15.51 (0.14) ^bB^	0.74 (0.05) ^bB^	1.38 (0.71) ^aB^	4.77 (0.03) ^cB^	0.12 (0.34) ^gA^	33.74 (1.76)
**Extrudates**								
CE	0.92 (0.03) ^fA^	1.94 (0.06) ^hA^	16.91 (0.11) ^eA^	0.75 (0.04) ^fA^	1.74 (0.07) ^cA^	6.88 (0.14) ^hA^	0.00 ^-B^	29.14 (0.45)
R4E	1.67 (0.09) ^deA^	8.59 (0.05) ^fA^	18.11 (0.31) ^dA^	1.01 (0.03) ^cdeA^	1.31 (0.08) ^dA^	7.83 (0.08) ^efA^	0.00 ^-B^	38.52 (0.64)
MDR4E	1.53 (0.02) ^eA^	8.07 (0.06) ^gA^	18.39 (0.23) ^dA^	0.93 (0.05) ^defA^	1.35 (0.13) ^dA^	7.48 (0.23) ^fgA^	0.00 ^-B^	37.75 (0.72)
RMDR4E	1.56 (0.07) ^eA^	8.05 (0.03) ^gA^	18.53 (0.26) ^cdA^	0.86 (0.03) ^efA^	1.41 (0.27) ^dA^	7.66 (0.13) ^efgA^	0.00 ^-B^	38.07 (0.79)
PPR4E	1.89 (0.05) ^dA^	9.01 (0.03) ^eA^	19.59 (0.51) ^cA^	1.11 (0.28) ^cdA^	1.90 (0.23) ^bcA^	8.04 (0.16) ^eA^	0.00 ^-B^	41.54 (1.26)
CDR4E	1.52 (0.26) ^eA^	8.27 (0.09) ^fgA^	18.43 (0.59) ^dA^	0.92 (0.34) ^defA^	1.34 (0.22) ^dA^	7.40 (0.03) ^gA^	0.00 ^-B^	37.88 (1.53)
R8E	2.95 (0.08) ^aA^	13.49 (0.40) ^bA^	21.88 (0.16) ^bA^	1.40 (0.07) ^abA^	2.00 (0.34) ^bA^	9.74 (0.21) ^abA^	0.00 ^-B^	51.46 (1.26)
MDR8E	2.86 (0.05) ^bA^	12.61 (0.03) ^cA^	21.00 (0.34) ^bA^	1.22 (0.05) ^bcA^	1.80 (0.18) ^bcA^	9.17 (0.08) ^cdA^	0.00 ^-B^	48.66 (0.73)
RMDR8E	2.85 (0.07) ^bA^	12.27 (0.25) ^cdA^	21.19 (0.52) ^bA^	1.17 (0.08) ^bcA^	1.94 (0.22) ^bA^	8.90 (0.13) ^dA^	0.00 ^-B^	48.32 (1.27)
PPR8E	3.31 (0.03) ^bA^	13.93 (0.06) ^aA^	23.02 (0.78) ^aA^	1.59 (0.06) ^aA^	2.45 (0.13) ^aA^	10.14 (0.13) ^aA^	0.00 ^-B^	54.44 (1.19)
CDR8E	2.55 (0.21) ^cA^	12.17 (0.23) ^dA^	21.13 (0.13) ^bA^	1.16 (0.24) ^cA^	1.85 (0.89) ^bcA^	9.44 (0.17) ^bcA^	0.00 ^-B^	48.30 (1.87)

Small different letters in superscript within column indicates significant changes between samples, by Fisher test (*p* < 0.05), comparing studied samples in mixtures or extrudates. Big different letters within a column indicate significant changes between samples, by Fisher test (*p* < 0.05), comparing mixtures and extrudates. R, rosehip; MDR, maltodextrin rosehip; RMDR, resistant maltodextrin rosehip; PPR, pea protein rosehip; CDR, cyclodextrin rosehip. 4, the concentration of 4% of rosehip preparation; 8, the concentration of 8% of rosehip preparation. M, mixture; E, extrudate.

## Data Availability

The datasets generated for this study are available on request to the corresponding author.

## Supplementary Materials

The following are available online at https://www.mdpi.com/article/10.3390/foods10102401/s1, Table S1. Mean values (and standard deviations) of phenolic acids (µg/gdry weight) of corn mixtures and extrudates, Table S2. Mean values (and standard deviations) of hydroxybenzoic acid (Di-Gallic acid) and flavanols content (µg/gdry weight) of corn mixtures and extrudates.
