# Supplementary material for: Effect on Nutritional and Functional Characteristics by Encapsulating Rose canina Powder in Enriched Corn Extrudates
Source: Foods. 2021 Oct 11;10(10):2401. doi: 10.3390/foods10102401 (PMC8535322; doi:10.3390/foods10102401)
Supplement: Supplementary file 1 [file foods-10-02401-s001.zip › foods-1399699-supplementary.pdf]

Table S1. Mean values (and standard deviations) of phenolic acids (µg/g$_{dry\ weight}$) of corn mixtures and extrudates.

Samples	Caffeic Acid	Siringic Acid	p-Coumaric Acid	Ferulic Acid	Di-caff	Total Phenolic Acids
			**Mixtures**			
CM	20.84 (0.06)^{aA}	14.26 (0.07)^{aA}	64.75 (0.21)^{aA}	42.86 (0.04)^{aA}	58.61 (0.06)^{aA}	201.32 (0.44)^{aA}
R4M	18.44 (0.26)^{bA}	13.31 (0.08)^{bA}	61.95 (0.05)^{bA}	31.30 (0.09)^{bB}	44.47 (0.03)^{bB}	169.48 (0.78)^{bB}
MDR4M	17.99 (0.03)^{cA}	13.22 (0.02)^{bcA}	61.56 (0.03)^{dA}	31.00 (0.12)^{dB}	44.22 (0.51)^{bB}	167.98 (0.71)^{cdB}
RMDR4M	18.10 (0.32)^{cA}	13.14 (0.06)^{cA}	61.21 (0.02)^{eA}	30.83 (0.22)^{eB}	44.09 (0.05)^{bB}	167.38 (0.67)^{eB}
PPR4M	18.14 (0.08)^{bcA}	13.16 (0.28)^{cA}	61.76 (0.06)^{cA}	31.13 (0.05)^{cB}	44.27 (0.03)^{bB}	168.46 (0.50)^{cB}
CDR4M	18.10 (0.20)^{cA}	13.19 (0.02)^{bcA}	61.54 (0.04)^{dA}	30.82 (0.34)^{eB}	44.09 (0.09)^{bB}	167.73 (0.69)^{deB}
R8M	17.34 (0.07)^{dA}	12.91 (0.08)^{dA}	59.55 (0.13)^{fgA}	29.49 (0.13)^{fB}	42.93 (0.02)^{cdB}	162.22 (0.43)^{fB}
MDR8M	17.20 (0.13)^{dA}	12.77 (0.05)^{eA}	59.56 (0.02)^{fgA}	29.22 (0.07)^{gB}	42.84 (0.08)^{cdB}	161.58 (0.35)^{gB}
RMDR8M	17.12 (0.05)^{dB}	12.62 (0.16)^{efA}	59.40 (0.15)^{ghA}	29.03 (0.08)^{hB}	42.74 (0.02)^{dB}	160.90 (0.46)^{hB}
PPR8M	17.02 (0.09)^{dB}	12.32 (0.03)^{fA}	59.38 (0.09)^{hA}	29.05 (0.23)^{hB}	43.34 (0.84)^{cB}	161.10 (1.28)^{ghB}
CDR8M	17.11 (0.08)^{dB}	12.65 (0.05)^{gA}	59.59 (0.03)^{fA}	28.99 (0.78)^{hB}	42.70 (0.15)^{dB}	161.05 (1.09)^{ghB}
			**Extrudates**			
CE	18.46 (0.22)^{aB}	12.43 (0.04)^{aB}	60.36 (0.05)^{aB}	32.52 (0.23)^{dB}	46.79 (0.40)^{eB}	170.56 (0.94)^{fA}
R4E	18.28 (0.07)^{abA}	11.86 (0.07)^{bB}	57.73 (0.13)^{bB}	37.92 (0.24)^{aA}	53.56 (0.12)^{aA}	179.35 (0.63)^{aA}
MDR4E	18.19 (0.08)^{abcA}	11.63 (0.02)^{cdB}	57.75 (0.07)^{bB}	37.76 (0.08)^{abA}	53.31 (0.13)^{aA}	178.65 (0.38)^{abA}
RMDR4E	18.09 (0.06)^{abcdeA}	11.69 (0.07)^{cB}	57.76 (0.04)^{bB}	37.78 (0.08)^{abA}	53.26 (0.22)^{abA}	178.58 (0.47)^{abA}
PPR4E	18.14 (0.09)^{abcdA}	11.59 (0.02)^{deB}	57.48 (0.11)^{cB}	37.81 (0.07)^{aA}	53.31 (0.33)^{aA}	178.33 (0.62)^{bA}
CDR4E	18.01 (0.08) ^{abcdeA}	11.45 (0.04)^{fgB}	57.36 (0.15)^{dB}	37.60 (0.09)^{bA}	52.71 (0.66)^{bA}	177.13 (1.02)^{cA}
R8E	18.24 (0.60)^{abcA}	11.51 (0.22)^{efB}	55.35 (0.04)^{fB}	36.62 (0.13)^{cA}	51.91 (0.07)^{cA}	173.63 (0.52)^{dA}
MDR8E	17.63 (0.16)^{eA}	11.47 (0.03)^{fgB}	55.37 (0.08)^{efB}	36.65 (0.22)^{cA}	51.63 (0.23)^{cdA}	172.74 (0.72)^{deA}
RMDR8E	17.67 (0.15)^{deA}	11.38 (0.07)^{gB}	55.46 (0.07)^{eB}	36.73 (0.31)^{cA}	51.50 (0.22)^{cdA}	172.75 (0.82)^{deA}
PPR8E	17.76 (0.02)^{cdeA}	11.47 (0.03)^{fB}	55.20 (0.15)^{gB}	36.54 (0.15)^{cA}	51.17 (0.07)^{dA}	172.14 (0.42)^{eA}
CDR8E	17.83 (0.03)^{bcdeA}	11.51 (0.12)^{efB}	55.41 (0.02)^{efB}	36.60 (0.06)^{cA}	51.43 (0.02)^{cdA}	172.78 (0.25)^{deA}

Small different letters in superscript within column indicates significant changes between samples, by Fisher test ($p < 0.05$) comparing studied samples in mixtures or extrudates. Big different letter within column indicates significant changes between samples, by Fisher test ($p < 0.05$) comparing mixtures and extrudates.

R, rosehip; MDR, maltodextrin rosehip; RMDR, resistant maltodextrin rosehip; PPR, pea protein rosehip; CDR, ciclodextrin rosehip. 4, concentration of 4 % of rosehip preparation; 8, concentration of 8 % of rosehip preparation. M, mixture; E, extrudate.

•

Table S2. Mean values (and standard deviations) of hydroxybenzoic acid (Di-Gallic acid) and flavanols content (μg/g$_{dry\ weight}$) of corn mixtures and extrudates.

Samples	Di-Gall	Procyan d1	Procyan d2	Cat	Q-acet-rham	I-glucur	Q-gluc	Q-glu-gluc-rham	I-gluc	I-acet-gluc-gluc	Q	Total Flavonoids
**Mixtures**												
CM	-^{k}	-^{k}	-^{j}	-^{k}	-^{h}	-^{i}	-^{k}	-^{h}	-^{i}	-^{j}	-^{k}	-^{k}
R4M	187.14 (0.41)^{gB}	38.62 (0.04)^{gA}	23.21 (0.06)^{fA}	182.44 (0.07)^{gA}	20.92 (0.04)^{fA}	13.74 (0.08)^{fA}	18.29 (0.03)^{gA}	18.33 (0.08)^{dB}	19.96 (0.08)^{eB}	17.62 (0.12)^{gB}	15.98 (0.25)^{fA}	558.11 (1.26)^{fA}
MDR4M	146.19 (0.32)^{iA}	37.34 (0.02)^{hA}	22.80 (0.15)^{gA}	183.14 (0.12)^{fA}	23.51 (0.02)^{eA}	13.73 (0.05)^{fA}	16.35 (0.07)^{jB}	18.00 (0.09)^{eB}	20.26 (0.50)^{eB}	14.93 (0.02)^{iB}	14.93 (0.08)^{jA}	515.18 (1.44)^{hA}
RMDR4M	124.43 (0.07)^{jA}	25.25 (0.03)^{jA}	22.63 (0.04)^{ghA}	123.44 (0.12)^{jA}	20.55 (0.05)^{gA}	12.84 (0.23)^{hA}	18.27 (0.04)^{gB}	16.67 (0.05)^{gB}	18.29 (0.45)^{gB}	18.90 (0.07)^{fA}	15.32 (0.03)^{iA}	416.59 (1.04)^{jA}
PPR4M	194.90 (0.06)^{fB}	27.1 (0.3)^{iA}	22.14 (0.07)^{iA}	131.05 (0.04)^{iA}	20.56 (0.03)^{gA}	13.34 (0.02)^{gA}	19.23 (0.02)^{fB}	16.62 (0.02)^{gB}	14.66 (0.02)^{hB}	16.29 (0.34)^{hB}	15.64 (0.02)^{hA}	491.46 (0.92)^{iA}
CDR4M	175.02 (0.06)^{hB}	40.70 (0.04)^{fA}	22.41 (0.13)^{hA}	165.04 (0.02)^{hA}	20.55 (0.09)^{gA}	13.72 (0.09)^{fA}	17.59 (0.05)^{iB}	17.59 (0.55)^{fB}	19.51 (0.34)^{fB}	17.93 (0.77)^{gB}	16.59 (0.33)^{gA}	526.65 (2.47)gA
R8M	736.79 (0.69)^{aA}	148.0 (0.3)^{aA}	205.2 (0.2)^{aA}	361.40 (0.02)^{aA}	31.98 (0.04)^{aA}	21.23 (0.08)^{aA}	44.40 (0.04)^{aA}	28.42 (0.08)^{bB}	32.22 (0.09)^{aB}	27.77 (0.08)^{aB}	23.49 (0.04)^{eA}	1660.86 (1.69)^{aA}
MDR8M	643.42 (0.13)^{bA}	120.60 (0.08)^{bA}	204.2 (0.2)^{bA}	319.04 (0.05)^{cA}	29.05 (0.03)^{cA}	20.49 (0.05)^{cA}	39.83 (0.03)^{bA}	31.67 (0.04)^{aA}	29.60 (0.11)^{cA}	26.75 (0.55)^{bB}	29.05 (0.52)^{aA}	1493.64 (1.84)^{bA}
RMDR8M	496.38 (0.28)^{eA}	80.9 (0.3)^{eA}	120.42 (0.03)^{eA}	287.65 (0.02)^{eA}	30.66 (0.03)^{bA}	20.79 (0.06)^{bA}	37.83 (0.08)^{cA}	28.41 (0.09)^{bB}	29.60 (0.05)^{cB}	25.80 (0.09)^{dB}	28.68 (0.37)^{bA}	1187.11 (1.37)^{eA}
PPR8M	507.93 (0.14)^{dA}	116.17 (0.08)^{cA}	168.61 (0.04)^{cA}	325.95 (0.37)^{bA}	28.99 (0.54)^{cA}	14.67 (0.08)^{eA}	37.12 (0.09)^{dA}	31.59 (0.03)^{aB}	28.98 (0.03)^{dB}	26.39 (0.05)^{cB}	27.02 (0.65)^{cA}	1313.42 (2.10)^{dA}
CDR8M	565.81 (0.21)^{cA}	108.88(0.02)^{dA}	155.16 (0.02)^{dA}	317.55 (0.02)^{dA}	24.42 (0.13)^{dA}	14.96 (0.02)^{dA}	29.98 (0.03)^{eB}	23.78 (0.02)^{cB}	30.31 (0.02)^{bA}	24.44 (0.03)^{eA}	26.07 (0.04)^{dA}	1321.36 (0.56)^{cA}
**Extrudates**												
CE	-^{j}	-	-	-	-	-	-^{k}	-^{j}	-^{g}	-^{j}	-	-^{k}
R4E	211.76 (0.34)^{eA}	n.d.	n.d.	n.d.	n.d.	n.d.	17.02 (0.02)^{iB}	31.66 (0.07)^{eA}	29.13 (0.04)^{eA}	19.83 (0.03)^{gA}	0.00^{B}	309.40 (0.5)^{fB}
MDR4E	116.65 (0.02)^{hB}	n.d.	n.d.	n.d.	n.d.	n.d.	19.56 (0.05)^{hA}	27.11 (0.55)^{hA}	26.01 (0.06)^{fA}	16.12 (0.02^{iA}	0.00^{B}	205.46 (0.7)^{iB}
RMDR4E	113.74 (0.73)^{iB}	n.d.	n.d.	n.d.	n.d.	n.d.	23.59 (0.23)^{gA}	28.27 (0.03)^{fgA}	27.97 (0.38)^{eA}	16.46 (0.04)^{iB}	0.00^{B}	210.03 (1.41)^{iB}
PPR4E	221.73 (0.18)^{dA}	n.d.	n.d.	n.d.	n.d.	n.d.	25.96 (0.34)^{eA}	36.80 (0.78)^{cA}	31.47 (0.78)^{dA}	23.97 (0.65)^{fA}	0.00^{B}	339.93 (2.39)^{dB}
CDR4E	196.45 (0.08)^{fA}	n.d.	n.d.	n.d.	n.d.	n.d.	28.54 (0.04)^{dA}	27.82 (0.03)^{ghA}	28.45 (0.56)^{eA}	18.85 (0.05)^{hA}	0.00^{B}	300.03 (0.76)^{hB}
R8E	339.13 (0.34)^{bB}	n.d.	n.d.	n.d.	n.d.	n.d.	33.09 (0.08)^{bB}	39.56 (0.22)^{bA}	40.05 (0.22)^{bA}	34.30 (0.67)^{bA}	0.00^{B}	486.15 (1.53)^{bB}

Table S2. *Cont.*

**Samples**	**Di-Gall**	**Procyan d**	Procyan d	Cat	Q-acet-rham	I-glucur	Q-gluc	Q-glu-gluc-rham	I-gluc	I-acet-gluc-gluc	**Q**	**Total flavonoids**
**Extrudates**												
MDR8E	196.82 (0.17)^{fB}	n.d.	n.d.	n.d.	n.d.	n.d.	25.35 (0.06)^{fB}	24.43 (0.19)^{iB}	29.09 (0.34)^{eA}	28.78 (0.04)^{cA}	0.00^{B}	304.46 (0.8)^{gB}
RMDR8E	193.62 (0.04)^{gB}	n.d.	n.d.	n.d.	n.d.	n.d.	25.09 (0.45)^{fB}	34.76 (0.34)^{dA}	34.70 (0.05)^{cA}	26.65 (0.76)^{dA}	0.00^{B}	314.81 (1.64)^{eB}
PPR8E	369.69 (0.02)^{aB}	n.d.	n.d.	n.d.	n.d.	n.d.	36.92 (0.57)^{aA}	43.47 (0.67)^{aA}	45.55 (1.06)^{aA}	37.78 (0.99)^{aA}	0.00^{B}	533.40 (0.75)^{aB}
CDR8E	333.02 (0.09)^{cB}	n.d.	n.d.	n.d.	n.d.	n.d.	31.60 (0.03)^{cA}	28.81 (0.94)^{fA}	25.38 (0.02)^{fB}	23.86 (0.05)^{fB}	0.00^{B}	442.68 (0.23)^{cB}

Small different letters in superscript within column indicates significant changes between samples, by Fisher test ($p < 0.05$) comparing studied samples in mixtures or extrudates. Big different letter within column indicates significant changes between samples, by Fisher test ($p < 0.05$), comparing mixtures and extrudates. R, rosehip; MDR, maltodextrin rosehip; RMDR, resistant maltodextrin rosehip; PPR, pea protein rosehip; CDR, ciclodextrin rosehip. 4, concentration of 4 % of rosehip preparation; 8, concentration of 8 % of rosehip preparation. M, mixture; E, extrudate. Di-Gall: Di-Gallic acid; Procyan d1: Procyanidin dimmer 1; Procyan d2: Procyanidin dimmer 2; Cat: Catechin; Q-acet-rham: Quercetin-acetyl-rhamnoside; I-glucur: Isorhamnetin-glucuronide; Q-gluc: Quercetin-glucoside; Q-glu-gluc-rham: Quercetin-glucosyl-glucosyl-rhamnoside; I-gluc: Isorhamnetin-glucoside; I-acet-gluc-gluc: Isorhamnetin-acetyl-glucosyl-glucoside; Q: Quercetin; n.d. – not detected.
